# Selection, engineering, and in vivo testing of a human leukocyte antigen–independent T-cell receptor recognizing human mesothelin

**DOI:** 10.1371/journal.pone.0301175

**Published:** 2024-04-04

**Authors:** Martyn J. Hiscox, Alexandra Wasmuth, Chris L. Williams, Jaelle N. Foot, Guy E. Wiedermann, Valeria Fadda, Sara Boiani, Terri V. Cornforth, Karolina A. Wikiert, Shaun Bruton, Neil Cartwright, Victoria Elizabeth Anderson, Christopher S. Barnes, Joao V. Vieira, Ian Birch-Machin, Andrew B. Gerry, Karen Miller, Nicholas J. Pumphrey

**Affiliations:** Research, Adaptimmune, Abingdon, Oxfordshire, United Kingdom; CCAC, UNITED STATES

## Abstract

**Background:**

Canonical α/β T-cell receptors (TCRs) bind to human leukocyte antigen (HLA) displaying antigenic peptides to elicit T cell−mediated cytotoxicity. TCR-engineered T-cell immunotherapies targeting cancer-specific peptide-HLA complexes (pHLA) are generating exciting clinical responses, but owing to HLA restriction they are only able to target a subset of antigen-positive patients. More recently, evidence has been published indicating that naturally occurring α/β TCRs can target cell surface proteins other than pHLA, which would address the challenges of HLA restriction. In this proof-of-concept study, we sought to identify and engineer so-called HLA-independent TCRs (HiTs) against the tumor-associated antigen mesothelin.

**Methods:**

Using phage display, we identified a HiT that bound well to mesothelin, which when expressed in primary T cells, caused activation and cytotoxicity. We subsequently engineered this HiT to modulate the T-cell response to varying levels of mesothelin on the cell surface.

**Results:**

The isolated HiT shows cytotoxic activity and demonstrates killing of both mesothelin-expressing cell lines and patient-derived xenograft models. Additionally, we demonstrated that HiT-transduced T cells do not require CD4 or CD8 co-receptors and, unlike a TCR fusion construct, are not inhibited by soluble mesothelin. Finally, we showed that HiT-transduced T cells are highly efficacious in vivo, completely eradicating xenografted human solid tumors.

**Conclusion:**

HiTs can be isolated from fully human TCR–displaying phage libraries against cell surface-expressed antigens. HiTs are able to fully activate primary T cells both in vivo and in vitro. HiTs may enable the efficacy seen with pHLA-targeting TCRs in solid tumors to be translated to cell surface antigens.

## Introduction

Antigen receptors on α/β T cells and B cells conventionally recognize distinct antigenic ligands, although they are generated by the same recombination system. Antigen receptors on B cells recognize conformational epitopes on native proteins, whereas canonical α/β T-cell antigen receptors (TCRs) recognize linear peptides of antigenic proteins bound in a groove of the major histocompatibility complex (MHC), or the human leukocyte antigen (HLA) in *Homo sapiens* [1]. This characteristic of TCRs is called MHC or HLA restriction and is programmed in the thymus during T-cell maturation [2].

**Fig 1:**
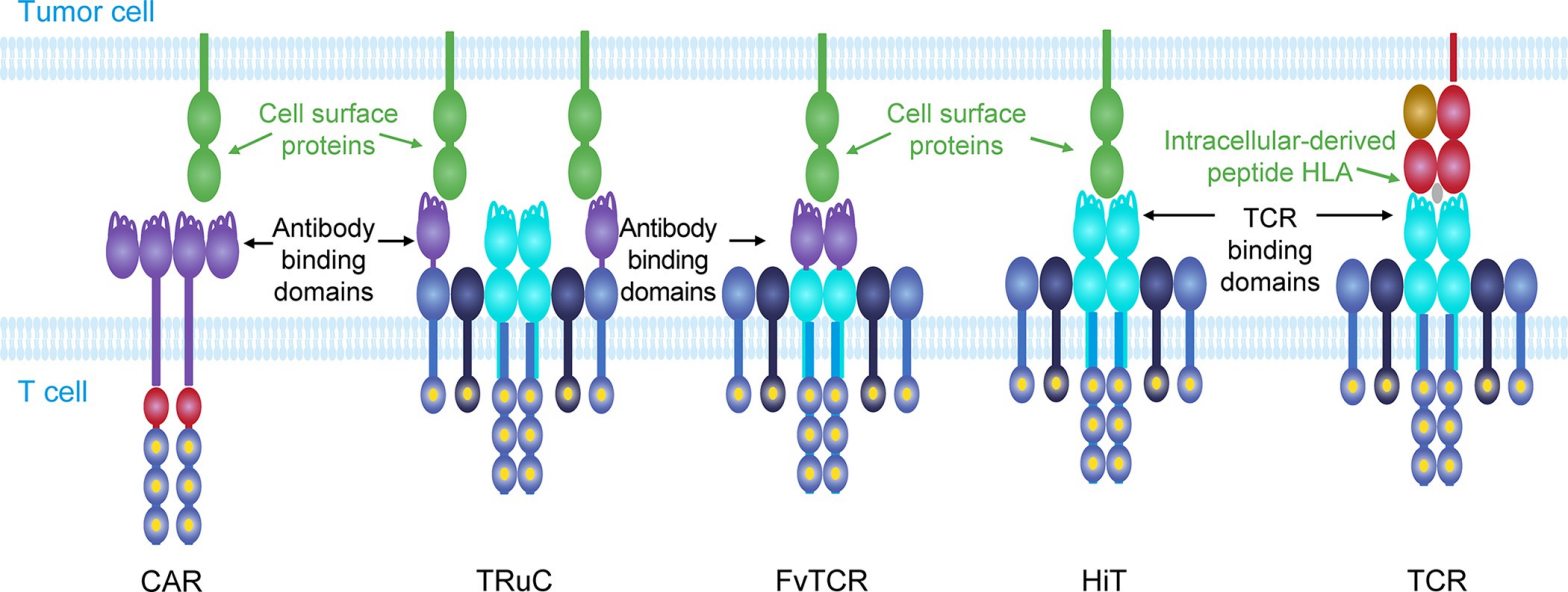
Immunomodulatory molecules that can be added to T cells for adoptive T-cell therapies. CAR T cells use an antibody fragment (such as a single-chain variable fragment as shown here) as the binding domain—which is fused to CD3ζ and often contains additional co-stimulatory domains—that targets a cell surface protein. TRuC T cells also use an antibody fragment (such as a single-domain antibody as shown here) that targets a cell surface protein; however, this antibody fragment is fused to another part of the TCR:CD3 complex—shown here, it is fused to the CD3ε, using natural T-cell signaling pathways. FvTCR T cells replace the variable domains of the TCR with the light and heavy chains of the antibody. HiT T cells use TCR binding domains to target cell surface proteins directly and therefore use natural cell signaling pathways. TCR T cells use a TCR binding domain to specifically target a peptide presented by the HLA molecule. CAR, chimeric antigen receptor; FvTCR, variable fragment T-cell receptor; HiT, human leukocyte antigen–independent T-cell receptor; HLA, human leukocyte antigen; TCR, T-cell receptor; TRuC, T-cell receptor fusion construct.

Most TCRs reported in the literature have been classified as HLA dependent (Fig 1). However, a small number of TCRs do not recognize the classical peptide-HLA (pHLA) target [3–13]. The identification of HLA-independent TCRs (HiTs) has generally been the result of investigations into the properties of TCRs isolated by repeated stimulations of peripheral blood mononuclear cells with cancerous cell lines or modified antigen-expressing cell lines [7,8,10].

An alternative strategy using mice has been demonstrated by the isolation of HiTs targeting CD48, CD102, and CD155 [11–13]. This strategy uses “quad-deficient” mice (*H2-Ab*^*−/−*^*B2m*^*−/−*^*Cd4*^*−/−*^*Cd8*^*−/−*^) with T cells lacking MHC and co-receptor proteins, and which also express the pro-survival *Bcl2* transgene so that traditional thymic selection is absent. Stimulation of T-cell hybridomas by plate-bound anti-TCRβ/anti-CD28 antibodies and MHC knockout antigen-presenting cells results in the isolation of HiTs [11–13].

The adoptive transfer of effector T cells recognizing tumor antigens is a well-established cancer immunotherapy strategy, efficacious in multiple indications [14,15]. The expansion of cells in vitro from patient-derived tumor-reactive T cells has shown promise in the clinic [16]. However, the efficacy of these therapies is often limited because most tumor antigens are self-like, and thymic selection removes TCRs that bind self-antigens. In addition, the levels of pHLA on the surface of some tumor cells can be low [17]. To overcome these problems, TCR affinity can be increased while maintaining specificity [18], thus improving tumor cell recognition and killing in vitro and in vivo [19–22], which leads to improved clinical efficacy across a range of tumor indications [23–25]. A key limitation of using HLA-dependent TCRs is that the HLA locus demonstrates polymorphism and therefore TCRs will typically be restricted to a specific HLA subtype [26]. The use of HiTs to target surface-expressed tumor-associated antigens would therefore enable the treatment of a greater proportion of patients.

In this proof-of-concept study, we utilized our phage display libraries presenting naive human α/β TCR repertoires to isolate a HiT against the tumor-associated antigen mesothelin. This is the first demonstration in the literature of an attempt to isolate a HiT against a defined target rather than post hoc determination of the target. Mesothelin is a 40-kDa cell surface glycoprotein [27] that is highly expressed in mesothelioma, pancreatic, ovarian, and other cancers but has limited expression in normal tissue, making it a promising target for tumor-specific therapy [28], including chimeric antigen receptor (CAR) T-cell therapy as detailed in a review by Klampatsa et al. [29].

## Materials and methods

### Library production and biopanning

To produce phage display libraries, purified messenger RNA (mRNA) from peripheral leukocytes from in-house blood donations was converted into complementary DNA using SMARTScribe reverse transcriptase (Takara). This complementary DNA was then amplified with primers corresponding to either TRAV12-2 or TRAV21 along with TRBV6 (because these chain pairings work well in our phage display system) and a primer corresponding to the constant domain of either TRAC or TRBC. These variable domains were then linked to the corresponding constant domains before shuffling of the α and β chains together to create artificial pairings to increase diversity and ligation into a phage display vector, creating two libraries: one TRAV12-2 TRBV6 and the other TRAV21 TRBV6.

Magnetic streptavidin-coated beads (M-280, Thermo Fisher Scientific) were coated with biotinylated recombinant mesothelin (ACRO Biosystems). The beads were incubated with 10^12^ plaque forming units per milliliter of TCR-displaying phage, followed by washing with powdered milk solution (3% w/v) and 0.1% Tween 20 in phosphate-buffered saline (PBS; Sigma) followed by elution. The eluate was used to infect fresh *Escherichia coli* TG2 cells at mid-log phase for 30–60 min at 37°C without shaking. After infection, the TG2 cells were plated on tryptone yeast extract and glucose plates containing ampicillin (100 μg/mL) for incubation overnight. After two rounds of panning, single clones were sequenced and clones with high convergence tested for binding.

### Protein expression and purification

The DNA sequences encoding parental and mutant TCR α and β chains, previously modified to promote formation of soluble disulfide-stabilized heterodimers during refolding [30], were inserted into a pGMT7-derived expression vector using polymerase chain reaction cloning.

TCR chains were expressed separately as inclusion bodies from the T7 Express or Rosetta (DE3) *E*. *coli* strain, refolded, and purified as previously described [20,30,31], with minor modifications.

Refolds were dialyzed extensively against 10 mM Tris pH 8 at 4°C and purified using HiTrap Capto Q ImpRes 5 mL (GE Healthcare) and Superdex 200 Increase 10/300 GL (GE Healthcare) columns for equilibrium dissociation constant (K_D_) determination. Protein concentration was determined by measurement of OD_280_ using an extinction coefficient calculated from the sequence, and quality assessed by sodium dodecyl sulfate–polyacrylamide gel electrophoresis on Mini-PROTEAN TGX Stain-Free Precast Gels (Bio-Rad).

### Surface plasmon resonance

Surface plasmon resonance (SPR) analysis of purified TCRs for K_D_ determination was performed using a Biacore 3000 instrument and flow cells (GE Healthcare), as described [32]. Biotinylated human mesothelin was immobilized onto streptavidin-coupled CM5 sensor chips (approximately 300 response units per flow cell for K_D_ determination). The chips were blocked with biotin. Non-biotinylated truncated mesothelin constructs (MN1–MN4) were captured via interaction with biotinylated tris-NTA (Biotechrabbit) that had been immobilized on streptavidin-coupled CM5 sensor chips. Binding measurements for serial dilutions of each TCR were performed at 25°C in PBS (Sigma) at a flow rate of 5 or 10 μL/min, starting from the lowest analyte concentration. SPR binding data for parental and most daughter mutant TCRs were processed using equilibrium binding analysis, except for Mut6, Mut7, and Mut8 mutant TCRs, which were also processed using kinetic analysis owing to longer dissociation half-lives.

Mesothelin-specific equilibrium binding responses were determined for each concentration using BIAevaluation software (GE Healthcare). Specific equilibrium response levels were then plotted against TCR concentration using GraphPad Prism (GraphPad Software Inc., San Diego, CA, USA), and K_D_ values were calculated by fitting to the 1:1 Langmuir binding model. For kinetic analysis, chip and reagent preparation was conducted identically to equilibrium analysis, and the experiments were performed in the same way. The association constant (k_on_) and dissociation constant (k_off_) values were determined separately by fitting of the association and dissociation curves, respectively, assuming 1:1 Langmuir binding. The K_D_ could then be calculated using BIAevaluation software as the ratio k_off_/k_on_.

### Cell culture

All cell lines were cultured at 37°C under 5% CO_2_ in the appropriate medium containing 10% fetal bovine serum and penicillin-streptomycin (15070063, Thermo Fisher Scientific). Cell lines J.RT3-T3.5 (TIB-153), HEK293T (CRL-3216), HCT116 (CCL-247), C32TG(CRL-1579), HepG2 (HB-8065), A375 (CRL-1619), SK-Br-3 (HTB-30), Cama-1 (HTB-21), HeLa (CCL-2), Capan-1 (HTB-79), Capan-2, (HTB-80) and K562 (CCL-243) were obtained from the American Type Culture Collection. DLD-1 (90102540) was obtained from the European Collection of Authenticated Cell Cultures. SNG-M (JCRB0179) was obtained from the Japanese Collection of Research Bioresources Cell Bank. The Jurkat TCR/CD3 effector cell line (J129A) was obtained from Promega. All cell lines were maintained in an appropriate medium under conditions recommended by the supplier. Patient-derived xenograft (PDX) cells were obtained from CrownBio. PDX cells were thawed as per manufactures guidelines and seeded directly into assay plates.

### Jurkat activation testing

J.RT3-T3.5 2B9 cells (1 million) were transduced by direct addition of 1 mL HEK293T supernatant (see S1 Methods) and incubated for 72 h. Transduced J.RT3-T3.5 2B9 cells and co-cultures of these cells with antigen-presenting cells (100,000 cells each) were incubated in 96-well plates (Greiner Bio-one) for approximately 16 h. Supernatants containing Jurkat cells were removed and the cells pelleted. Cells were washed once with PBS with 0.1% fetal bovine serum (Gibco) before incubation with anti-CD69-PE (12-0699-42, Thermo Fisher Scientific), anti-CD3-APC-eFluor780 (47-0038-42, Thermo Fisher Scientific), and anti-CD8-VioBlue (130-110-684, Miltenyi Biotec) for 30 min at 4°C. Cells were pelleted and washed once with PBS with 0.1% fetal bovine serum. Cells were pelleted and resuspended in 7-AAD solution (51–6898, BD Pharmingen) before data were acquired on a Miltenyi MACSQuant flow cytometer. Data were analyzed using FlowJo v10.6.0 (FlowJo LLC). CD69-positive events were quantified upon gating on lymphocytes (SSC-A vs FSC-A), singlets (FSC-A vs FSC-H), live cells (7-AAD vs FSC-A), and CD8- and CD3-positive cells (S1 Fig).

### Primary T-cell transduction

For small-scale T-cell transduction, peripheral blood mononuclear cells were isolated from healthy donor-derived fresh blood and modified using lentivirus (produced via the large-scale method; S1 Methods), as previously described [19].

For large-scale T-cell transduction, CD3^+^ total nucleated cells were isolated from thawed, previously frozen Leukopak apheresis (HemaCare) using CD3/CD28 Dynabeads (43310D, Thermo Fisher Scientific). Cells were seeded into G-Rex 6M plates (80660M, Wilson Wolf), transduced using lentivirus the next day and harvested after 10 days.

### Interferon γ enzyme-linked immune absorbent spot

HiT activity was measured using interferon γ (IFN-γ) enzyme-linked immune absorbent spot (ELISpot) (551873, 557630, and 551951; BD Biosciences), following the manufacturer’s instructions. T cells and target cells were each seeded at 50,000 cells per well and incubated on Multiscreen_HTS_ IP filter 0.45 μm plates (MSIPS4W10, Millipore) for approximately 17 h before assay development. Plates were imaged and analyzed using a Series 6 Ultra-V ImmunoSpot Analyzer (Cellular Technology). Spot-forming units were limited to a maximum of 500 spot-forming units per well, owing to signal saturation.

### Supernatant enzyme-linked immunosorbent assays

Target cells were seeded in 384-well microplates (781074, Greiner) at 8,000 cells per well except HeLa (4,000 cells per well). The following day, 10,000 T cells per well were added and co-cultures incubated for approximately 22 h before collection of supernatants.

For the PDX assay, target cells were seeded in 96-well Primaria microplates (353872, Corning) as follows: Capan-2 (40,000 cells per well, R10 medium), lung PDX 1 (50,000 cells per well, ACL4 medium), and lung PDX 2 (100,000 cells per well, ACL4 medium). The following day, ACL4 medium was washed out to R10 medium and 80,000 T cells per well were added and co-cultures incubated for approximately 24 h before collection of supernatants.

Human IFN-γ DuoSet and Human Granzyme B DuoSet enzyme-linked immunosorbent assay (ELISA) kits (DY285B and DY2906-05, respectively, R&D Systems) were used to assess IFN-γ and granzyme B release, respectively. Manufacturer protocols were followed, with minor modifications (see S1 Methods). Plates were developed using Glo Substrate Reagent Pack (DY993, R&D Systems) and read on a FLUOstar Omega plate reader (BMG Labtech) with OMEGA control software v.5.10 R2 (BMG Labtech).

### Cytotoxicity assay

SNG-M, HCT116, A375, and SK-BR-3 cells were each seeded as per supernatant ELISAs and allowed to form monolayers overnight before addition of 0.5 μM (final) IncuCyte Caspase-3/7 Green Dye for Apoptosis (4440, Sartorius), either with or without 10,000 T cells per well. For the PDX assay, target cells were seeded as per supernatant ELISAs. The following day, ACL4 medium was washed out to R10 medium and 0.5 μM (final) IncuCyte Caspase-3/7 Green Dye for Apoptosis (4440, Sartorius), either with or without 80,000 T cells per well.

Plates were imaged from this point using the phase and green fluorescence channels in the IncuCyte ZOOM system (Sartorius) with 10× objective lens at 2-h (3 h for PDX assays) repeating intervals. The number of caspase 3/7 positive cells (apoptotic target cells) per millimeter squared over time was enumerated for all conditions up to 72 h after T-cell addition using IncuCyte ZOOM 2018A software (Sartorius)—dying T cells were gated out by size exclusion.

### Co-receptor dependency assay

CD4-positive and CD8-positive populations were negative sorted with CD8 and CD4 MicroBeads (Miltenyi Biotec), respectively, and preincubated with anti-CD4 (clone RPA-T4, 300501, BioLegend), anti-CD8 (clone SK1, 344702, BioLegend), or isotype control (Mouse Igg1,κ, 400101, BioLegend) antibodies for 30 min. T cells (10,000 per well) were then cultured with mesothelin and MAGE-A4–positive (A375 transfected with MSLN; S1 Methods, 10,000 cells per well) target cells at an effector-to-target ratio of 1:1. IFN-γ secretion was assessed in the supernatant by ELISA after overnight incubation at 37°C and 5% CO_2_, according to the manufacturer’s instructions (R&D Systems). Data were analyzed using a two-way analysis of variance with Tukey correction for multiple comparisons in GraphPad Prism (GraphPad Software Inc., San Diego, CA, USA),

### Soluble mesothelin blocking assay

The TCR/CD3 effector cells (interleukin-2 [IL-2], Promega, 30,000 cells per well) transduced with different constructs were cultured with mesothelin-positive target cells (Capan-2) or mesothelin-negative target cells (K562) (10,000 cells per well) at an effector-to-target ratio of 3:1. The effector cells were preincubated with different amounts of recombinant mesothelin protein (range 0.04–40 μM) produced as above. After 6 h of incubation at 37°C and 5% CO_2_, the bioluminescent signal was detected with Bio-Glo™ Luciferase Assay System (G7940, Promega) and measured on a FLUOstar Omega reader (BMG Labtech).

### Capan-2 xenograft model

Immunodeficient NSG^®^ (NOD.Cg-*Prkdc*^*scid*^*II2rg*^*tm1Wjl*^/SzJ, Charles River Laboratories) mice (age 6–7 weeks) were inoculated subcutaneously with 1 × 10^7^ Capan-2 tumor cells in 50% Matrigel and, once tumors had grown to 100–200 mm^3^ (after 11 days), mice were randomized into treatment groups (n = 6–8 per group), with HiT or control T cells administered intravenously on day 0. Body weight and tumor volume (volume = 0.5[length × width^2^]) were recorded three times weekly throughout. Additional information per the guidelines Animal Research: Reporting of In Vivo Experiments 2.0 can be found in S1 Methods.

### Ethics approval

After obtaining written informed consent (recruitment period: August 22, 2019 to November 28, 2019), healthy donor T cells were used to produce large-scale or small-scale engineered T-cell batches. The study was approved by the South Central—Oxford A Research Ethics Committee (13/SC/0227). All methods were performed in accordance with relevant guidelines and regulations, and all mandatory laboratory health and safety procedures were complied with during the experimental work. All animal studies were performed in accordance with the UK Animals (Scientific Procedures) Act 1986 in line with Directive 2010/63/EU of the European Parliament and the Council of 22 September 2010, on the protection of animals used for scientific purposes.

## Results

### Isolation of a mesothelin-binding clone from a naive phage display library

**Fig 2:**
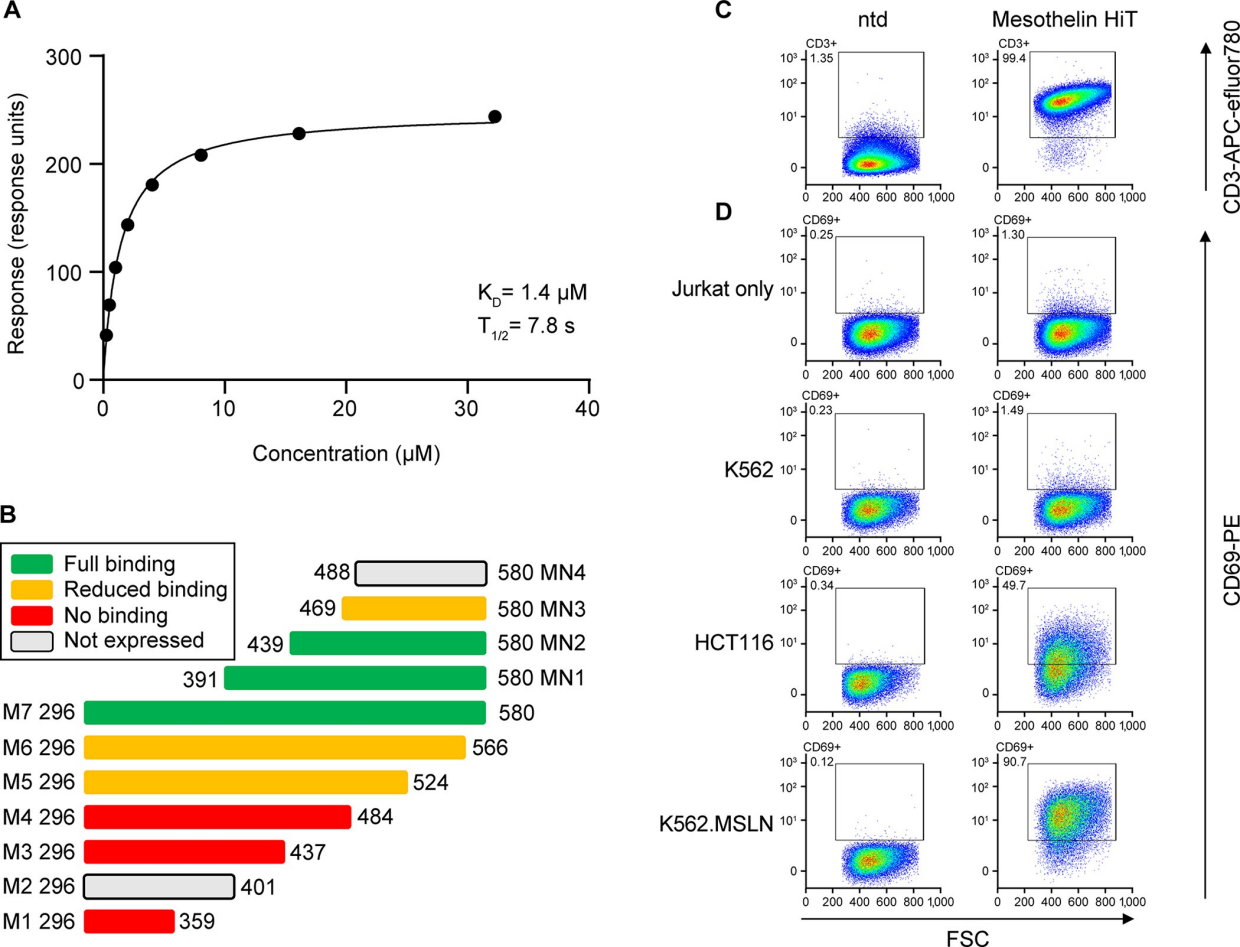
Initial characterization of a HiT against human mesothelin. (A) Representative example of surface plasmon resonance data. Equilibrium binding with a fit to a 1:1 Langmuir equation (one of four independent experiments is shown). (B) Epitope analysis of T-cell receptor binding to full-length (M7) and truncated (M1–6, MN1–4) mesothelin. Reduced binding is defined as a reduction in affinity (greater than five-fold increase in K_D_) or reduction in maximal binding response (maximal binding response less than 10% of the theoretical maximum based on the amount of immobilized mesothelin). (C) ntd 2B9 Jurkat T cells (left column) and mesothelin HiT–transduced Jurkat T cells (right column) were analyzed for CD3 expression, demonstrating successful transduction. (D) Jurkat T cells were cultured alone or co-cultured with antigen-positive (HCT116, K562.MSLN) or antigen-negative (K562) cell lines. CD69 surface expression was monitored by flow cytometry. HiT, human leukocyte antigen–independent T-cell receptor; K_D_, equilibrium dissociation constant; ntd, non-transduced; T_1/2_, dissociation half-life.

Naive phage display libraries expressing human α/β TCRs were subjected to two rounds of biopanning against recombinant biotinylated mesothelin. The DNA sequence of the TCRs isolated from the libraries was analyzed, and four converged TCRs were identified from two libraries. These TCRs were produced in soluble form and the binding was analyzed by SPR. Of these TCRs, one (with variable domain sequences as per S1 Data) showed antigen binding, with a mean ± SD (four independent experiments) K_D_ of 1.1 ± 0.4 μM, as calculated by equilibrium binding, and a dissociation half-life of 7.4 ± 0.6 s (Fig 2A and S2 Fig).

To further understand the interaction between the TCR and mesothelin, a series of truncated forms were produced. Of the 10 truncated forms designed, eight were successfully produced from HEK293T cells, whereas two were unable to be expressed and purified. Using SPR, these C-terminal truncations (M1–6, excluding M2) and N-terminal truncations (MN1–3) were compared alongside full-length mesothelin (M7) for their ability to bind to the TCR (S3 Fig). These data suggest that the TCR is binding an epitope between residues 439 and 580 (Fig 2B).

The affinity of the mesothelin HiT is at the upper limit of traditional pHLA:TCR binding interactions [33]. However, a sub- to low-micromolar affinity for the mesothelin HiT is in line with the affinities of two HiTs identified against murine CD155, at 0.23 and 0.28 μM [11,12], and a HiT that binds to murine CD102, at 0.5 μM [13].

### Mesothelin HiT–transduced Jurkat cells are activated by antigen-positive cell lines

Given the structural similarities between antibodies and TCRs, along with the similarities between antibodies and HiTs that bind to the same epitope [7], we anticipated that we would be able to identify a TCR that could bind a cell surface tumor-associated antigen, but it was unclear how well such a TCR would enable T-cell activation. When antibodies are generated and used to form CARs, the efficacy is often dependent on the position of the epitope and its distance from the cell surface [34,35]. The kinetic segregation model of T-cell activation [36] requires that the correct size of the immunological synapse is formed to exclude CD45 from the site of the TCR-ligand complex, resulting in net phosphorylation [37]. We therefore sought to determine whether this HiT could not only bind to mesothelin but also activate T cells when presented with a mesothelin-expressing target cell.

The commercially available Jurkat J.RT3-T3.5 cell line, which lacks expression of the TCR β chain, was transduced with a CD8-carrying lentivirus to yield the clone 2B9. 2B9 cells do not display a TCR at the cell surface and therefore are negative for CD3 when measured by flow cytometry but can be activated through introduced TCRs. 2B9 cells were subsequently transduced with a lentivirus containing the α/β TCR linked by a P2A skip sequence. Transduction levels were determined by measuring the percentage of cells that were CD3 positive, indicating transduction of the TCR and display at the surface of a TCR-CD3 complex.

2B9 cells transduced with the mesothelin HiT, along with non-transduced 2B9 cells, were co-cultured with antigen-positive and antigen-negative cells overnight (see Materials and methods; Jurkat activation testing). Cells were then stained for CD8, CD3, and CD69 to allow detection of 2B9 cells that had been transduced (Fig 2C) and those that were activated, which is shown by the presence of the early activation marker CD69 on the cell surface. Specific activation of the mesothelin HiT was detected when T cells were co-cultured with antigen-positive HCT116 cells, but no activation was detected in either non-transduced cells or transduced cells co-cultured with antigen-negative K562 cells (Fig 2D; full gating strategy in S1 Fig). These data indicate that the binding of the HiT to mesothelin on the cell surface can activate the T cell, presumably forming an immunological synapse at the correct distance to allow for CD45 exclusion.

To exclude the possibility that the mesothelin HiT was binding to a different cell surface epitope, K562 cells were stably transfected with human mesothelin linked by a P2A sequence to green fluorescent protein to produce K562.MSLN. Wild-type and K562.MSLN lines were co-cultured with the mesothelin HiT, and the level of activation was again measured by the presence of CD69 on the T-cell surface (Fig 2D). These data show that introduction of mesothelin to the K562 cell line allows for recognition by the mesothelin HiT and subsequent activation of the T cell. This demonstrates that activation is due to formation of a HiT-mesothelin immunological synapse, although the exact nature of this synapse is currently not known.

### Mesothelin HiT–transduced primary T cells kill antigen-positive cell lines and PDXs

**Fig 3:**
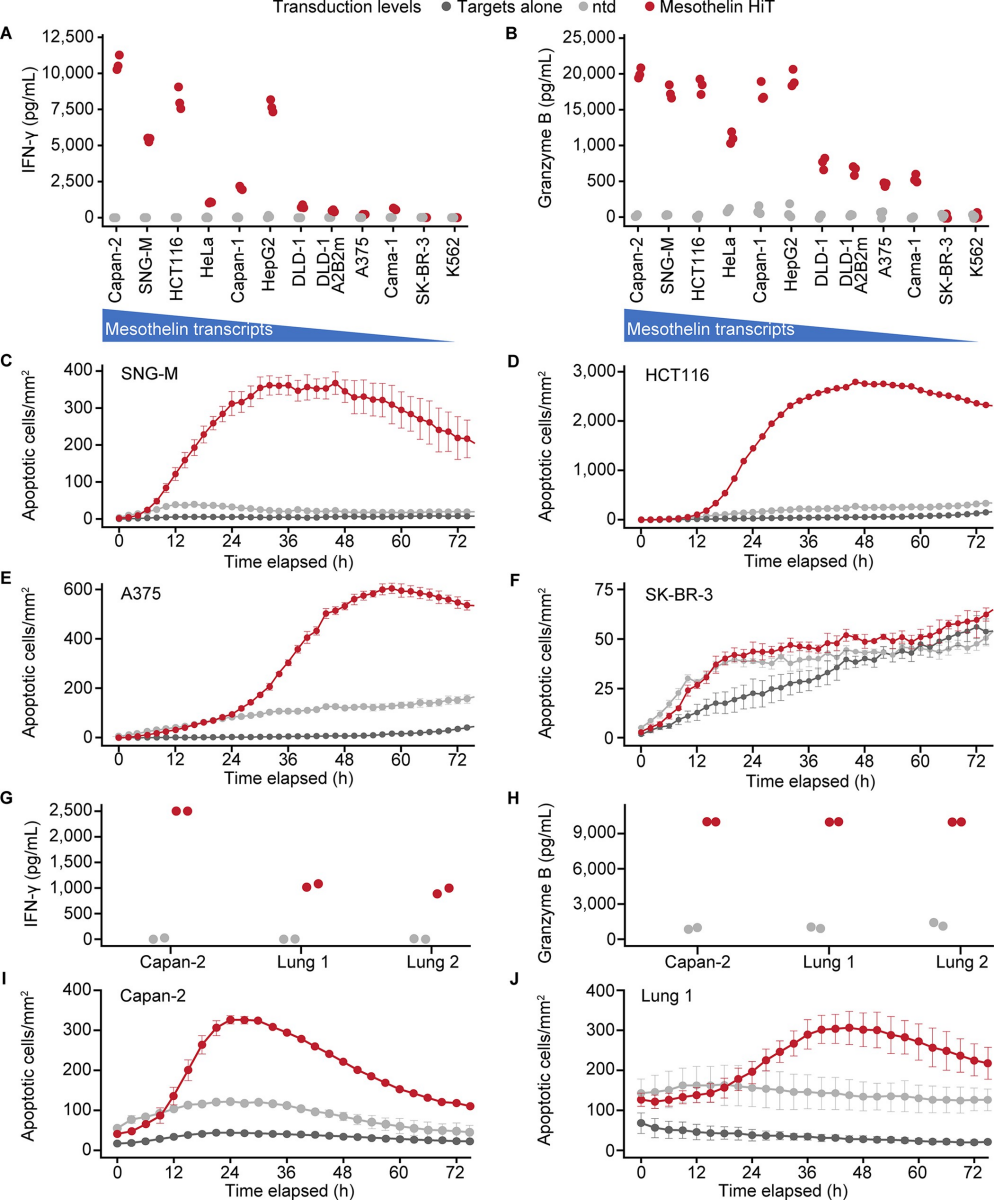
Primary T-cell activity of a HiT targeting human mesothelin. (A) IFN-γ and (B) granzyme B released by ntd T cells (gray) or mesothelin HiT T cells (red) from a representative donor are shown in triplicate against a panel of target cell lines (shown in descending order of mesothelin quantitative polymerase chain reaction score, from left to right). (C–F) Cytotoxic activity of the mesothelin HiT against target cell lines with varying mesothelin expression. The number of apoptotic target cells per millimeter squared over time is shown for (C) SNG-M, (D) HCT116, (E) A375, and (F) SK-BR-3 cells in monoculture (dark gray) or co-cultured with either ntd T cells (light gray) or mesothelin HiT T cells (red) from a representative donor. Mean and standard error of the mean are shown from three replicates for each condition/time point. (G) IFN-γ and (H) granzyme B released by ntd T cells (light gray) or mesothelin HiT T cells (red) from a different representative donor are shown in duplicate against Capan-2 cell lines and two lung patient-derived xenograft models that are positive for mesothelin. Cytotoxic activity of the mesothelin HiT against (I) Capan-2 and (J) a single lung patient-derived xenograft sample. The number of apoptotic target cells per millimeter squared over time is shown for cells in monoculture (dark gray) or co-cultured with either ntd T cells (light gray) or mesothelin HiT T cells (red) from a representative donor. Transduction efficiencies for the transduced T cells can be found in S2 Table. HiT, human leukocyte antigen–independent T-cell receptor; IFN-γ, interferon γ; ntd, non-transduced.

To confirm that the mesothelin HiT could induce cytotoxic activity, primary T cells from three healthy donors were transduced with lentiviral vectors encoding the HiT. A panel of antigen-positive and antigen-negative cell lines with a variety of different HLA class I alleles was chosen as per S1 Table, and the levels of mesothelin mRNA were measured using quantitative polymerase chain reaction (see S1 Methods). Of special interest are the cell lines SNG-M and DLD1, which express no surface HLA class I (S4 Fig). Cell lines and transduced T cells were co-cultured overnight before the supernatants were harvested and assayed for IFN-γ and granzyme B (Fig 3A and 3B). These results demonstrate that when transduced into T cells, the mesothelin HiT induces activation in a manner that correlates well with the mRNA level of the antigen-presenting cell. Secondly, the activity against cell lines with either no surface expression of HLA class I and the expression of a diverse population of HLA types across other cell lines increases the evidence that the activity seen in these T cells is not caused by cross-reactivity to a peptide-HLA, but rather by directing binding of the TCR to surface-displayed mesothelin.

To assess the ability of the mesothelin HiT–transduced T cells to kill tumor cell lines, a cytotoxicity assay was used, allowing direct visualization of the apoptosis of target cells in real time. Due to the size and density difference, comparisons can only be made between transduced T cells, non-transduced T cells, and the targets alone against the same cell line in a single experiment. This is shown in Fig 3C–3F, which demonstrates that co-cultured HiT-transduced T cells can specifically kill highly antigen-positive cells such as SNG-M and HCT116. However, there is no additional killing of antigen-negative SK-BR-3 above the background seen with targets alone or in the presence of non-transduced T cells. A375 cells have low expression of mesothelin and can be killed, albeit with a delay compared with SNG-M and HCT116 cells. This demonstrates the sensitivity of using natural T-cell signaling, which is known to require very few TCR-target contacts [38,39] and has been shown to be more sensitive than equivalent antibody binding domains in CAR T cells [40].

Following these results, primary T cells from two additional donors were prepared and co-cultured with the pancreatic Capan-2 cell line and with cells isolated from PDX models derived from patients with lung cancer and assayed as described earlier, demonstrating activation of T cells against these models (Fig 3G–3J). The T cells showed robust tumor cell killing of the in vitro PDX model, demonstrating the efficacy of the HiT in a more physiologically relevant system.

### Affinity modulation of mesothelin HiT allows for targeting of a therapeutic window

Autologous cell therapies are not without risk: off-target cross-reactivity can result in severe toxicities to patients and, in some cases, death [19]. On-target off-tumor reactivity has also proved dangerous when vital organs express small amounts of the target protein, which was either unknown or at a higher level than expected from preclinical testing [41–43]. For standard pHLA-targeting TCRs, it has been demonstrated that the affinity and specificity of TCRs can be fine-tuned to ensure they are not cross-reactive with pHLA that present a similar three-dimensional surface, thereby reducing the likelihood of off-target cross-reactivity [20]. However, affinity modulation of HiTs has not previously been shown. We sought to demonstrate that we could modulate the potency of our HiT to recognize discrete levels of mesothelin expression.

Complementarity-determining regions (CDRs) 1 and 2 of both the α and β TCR chains were mutated to prepare a panel of variants with both increased and decreased affinity. This panel was tested by SPR, CD69 activation, and ELISpot assays. Mutations in just two positions, α CDR1 S6 (a1_S6) and β CDR1 E4 (b1_E4), were able to produce a panel with approximately 10-fold higher and 10-fold lower affinity (Table 1).

**Table 1:** Binding characteristics of the parental mesothelin HiT and a panel of mutants, as determined by surface plasmon resonance from a single representative experiment.

HiT name	Mutation position	K_D_, μM	Dissociation half-life, s
Mut1	a1_S6	9.7	1
Mut2	a1_S6	8.3	2.4
Mut3	a1_S6	5.3	1.7
Mut4	a1_S6	3.2	2.5
Mut5	b1_E4	1.8	8
WT		1.4	7.8
Mut6	b1_E4	0.4	12.7
Mut7	b1_E4	0.1	Extending half-life >60 s
Mut8	a1_S6	0.1	26.5

HiT, human leukocyte antigen–independent T-cell receptor; K_D_, equilibrium dissociation constant.

In canonical pHLA-binding TCRs, CDR1 and CDR2 are often considered to bind the HLA [44], whereas CDR3 is considered to bind to the docked peptide [45,46]. However, the ability of TCR germline CDR1s and CDR2s to bind to a variety of different HLA alleles of both class I and class II suggests different binding modes are available to the TCR and HLA [47]. This ability to utilize different binding modes with different HLA alleles may allow the CDR1s and CDR2s of this mesothelin HiT to play an important role in antigen binding.

The mutant panel was subsequently transduced into 2B9 cells, as per the wild type, co-cultured with antigen-positive cells, and CD69 upregulation measured to ensure that none of the mutations resulted in the loss of T-cell activation (S5 Fig). A decrease in activation was evident in the three mutants with the lowest affinity (Mut1–Mut3) but all other mutants (Mut4–Mut 8) activated at the same level as the parental against the highly antigen-positive HCT116 target cells.

**Fig 4:**
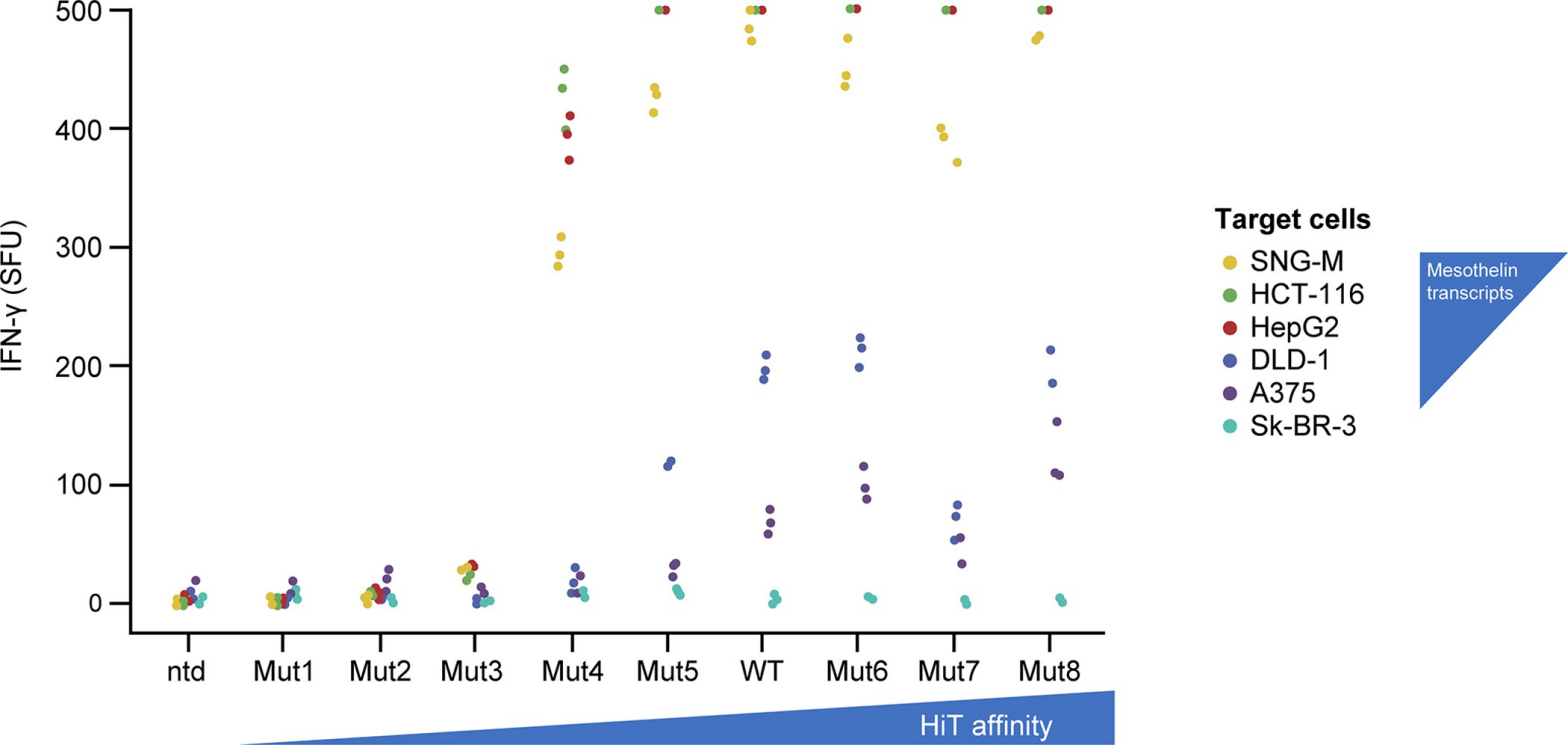
Relative activity of parental and engineered mesothelin HiTs against target cell lines by IFN-γ enzyme-linked immune absorbent spot. The activity of non-transduced T cells or T cells transduced with the parental HiT or eight engineered HiT mutants (shown in increasing affinity order, from left to right) was assessed against a panel of six target cell lines (shown on key in decreasing order of mesothelin quantitative polymerase chain reaction score from top to bottom). The number of IFN-γ SFU are shown from three replicates for each condition with a representative donor. SFU were limited to a maximum value of 500. Transduction efficiencies for the transduced T cells can be found in S2 Table. HiT, human leukocyte antigen–independent T-cell receptor; IFN-γ, interferon γ; ntd, non-transduced; SFU, spot-forming units; WT, wild type.

The parental and mutant HiT panel were subsequently transduced into primary T cells isolated from three healthy donors. The panel was then co-cultured with antigen-positive and antigen-negative cell lines with different levels of antigen expression, and the IFN-γ levels quantified by ELISpot assay (Fig 4). For the higher-expressing cell lines, little to no difference was seen for the five mutants with the highest affinity. The mutant with the fifth highest affinity (Mut4) showed a slight decrease in activity with the higher-expressing cell lines, with the next mutant (Mut3) showing an almost complete loss of activity with the higher-expressing cell lines. Unlike the Jurkat-based CD69 assay, no activity was measurable for the two lowest-affinity mutants. Analysis of the lower-expressing cell lines (such as A375) demonstrated that the higher-affinity mutants do elicit a stronger response as the affinity increases. However, Mut7 did not follow this trend, possibly owing to its extremely long dissociation half-life (>60 s) as an isolated protein.

These data demonstrate that modulation of HiT potency is achievable by engineering of the CDR1 and CDR2 regions of the HiT. Any first-in-human trials would need to be preceded by thorough analysis of the therapeutic window of mesothelin expression and subsequent affinity optimization, which falls outside of the scope of this manuscript.

### HiT T cells do not require either the CD4 or CD8 co-receptor

**Fig 5:**
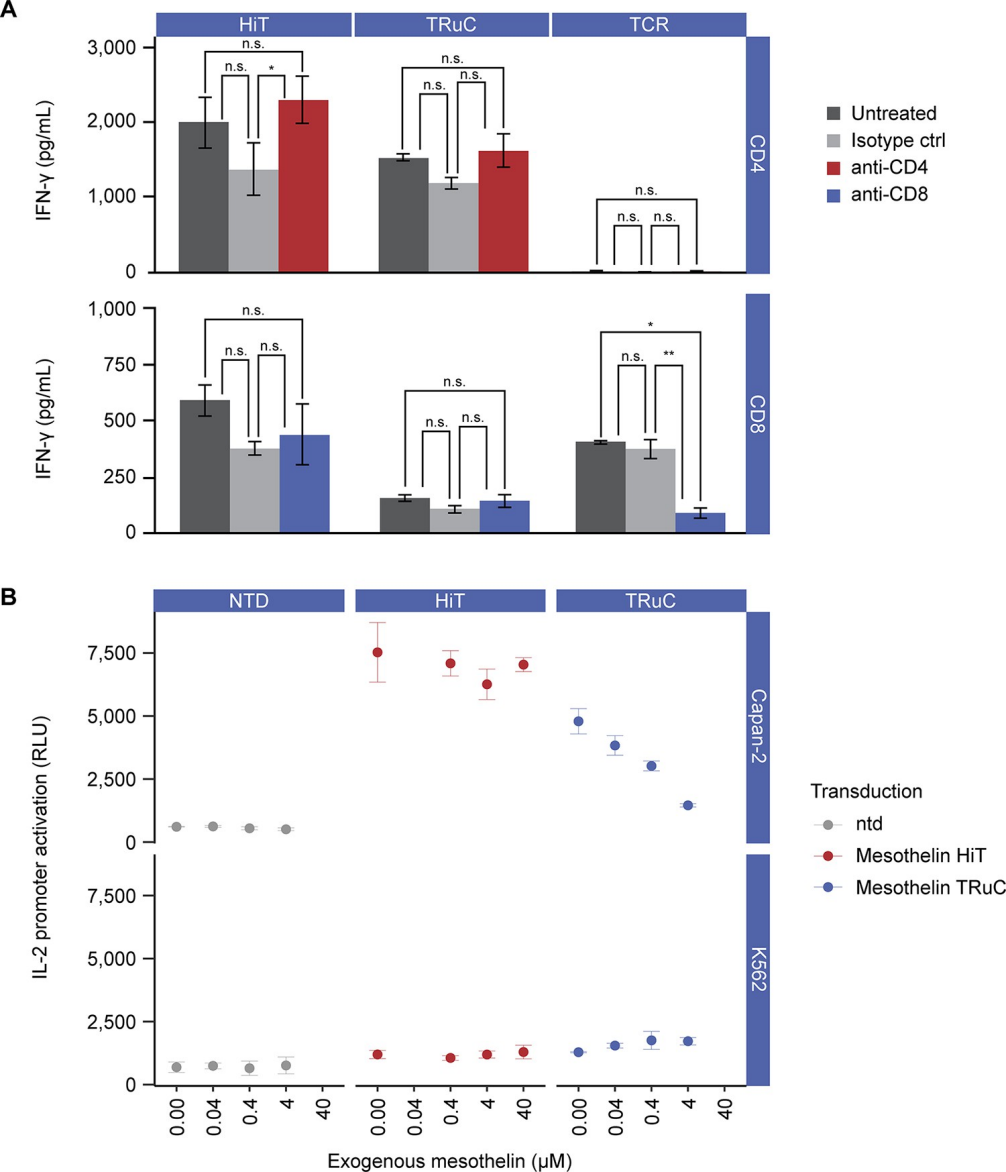
Mechanistic investigations of HiT function. (A) The activity of primary T cells in either the CD4 or CD8 subset were studied for the mesothelin HiT, a mesothelin TRuC, and an HLA-A02*01 displayed MAGE-A4 peptide targeting TCR against a MAGE-A4–positive and mesothelin–over-expressing A375 cell line. Before co-culture with target cells, CD4 or CD8 T cells were incubated with anti-CD4 or anti-CD8 antibodies, respectively; after overnight incubation, IFN-γ release into the supernatants was measured using enzyme-linked immunosorbent assay. Data were analyzed using a two-way analysis of variance n.s. >0.05 * p ≤ 0.05, ** p ≤ 0.01 (GraphPad Prism). (B) Inhibition of the mesothelin-targeting HiT and TRuC by soluble mesothelin was measured using a modified Jurkat cell line expressing luciferase from an IL-2 promoter (TCR/CD3 Effector Cells IL-2, Promega). A mesothelin-positive cell line (Capan-2) and a mesothelin-negative cell line (K562) were incubated with a range of mesothelin concentrations between 0.04 and 40 μM, before being cultured with effector cells. Activation of the IL-2 promoter on effector cells was detected at 6 h. Transduction efficiencies for the transduced T cells can be found in S2 Table. ctrl, control; HiT, human leukocyte antigen–independent T-cell receptor; IFN-γ, interferon γ; IL-2, interkeukin-2; n.s., non-significant; ntd, non-transduced; RLU, relative light units; TCR, T-cell receptor; TRuC, T-cell receptor fusion construct.

TCRs that bind to pHLA class I or class II utilize the CD8 or CD4 co-receptor, respectively, to enhance the recruitment of Lck to the TCR/CD3 complex [48]. We hypothesized that a truly HLA-independent TCR would not be dependent on either CD4 or CD8 and therefore should function in either subset of T cell, as is the case for antibody-based molecules such as CARs [49] and presumably for TCR fusion constructs (TRuCs). To test this hypothesis, a TRuC targeting mesothelin was prepared and cloned into our standard lentiviral vector. We chose to use a TRuC for these comparisons as these have shown favorable results in the clinic, and their use of TCR:CD3 complex for activation better match the mechanism of HiT activation when compared with the purely artificial manner of CAR-T activation. Subsequently, lentivirus and T cells were prepared using our in-house large-scale T-cell methods. This mesothelin-directed TRuC, our mesothelin-targeting HiT, and a TCR targeting an HLA-A02*01 presented MAGE-A4 peptide were co-cultured with A375 cells (which naturally express MAGE-A4) modified to overexpress mesothelin. IFN-γ release was assessed by ELISA in the presence or absence of antibodies blocking CD4 or CD8 (Fig 5A). These data show that the canonical class I TCR, as expected, is only able to activate in CD8 T cells and is inhibited when CD8 is blocked by an antibody. Both the HiT and TRuC show activity in both CD4 and CD8 T cells and are unaffected by the addition of blocking antibodies, demonstrating their HLA independence.

### HiT T cells are not inhibited by soluble mesothelin in vitro

One potential hurdle in targeting cell surface proteins is that soluble forms arising from either proteolytic shedding or splice variants that lack transmembrane or anchoring regions are often dysregulated in diseases such as cancer [50]. Accumulation of high levels of shed antigen in the tumor microenvironment can compete with cell surface antigen and is known to affect the activation of CARs [51,52]. To test if this phenomenon would affect activation of HiT T cells, we mimicked high levels of shed mesothelin in vitro [53]. Using a Jurkat cell line that links a luciferase reporter to an IL-2 promotor, the activation via the HiT and TRuC was tested in the presence of a titration of recombinant soluble mesothelin (Fig 5B). These results show that the TRuC, which uses a high-affinity (25 nM) antibody to target mesothelin, is affected at physiologically relevant levels [53] (0.04 μM) of soluble mesothelin. Activation of IL-2 signaling via the HiT, with its lower affinity, remains stable in the presence of up to 40 μM soluble mesothelin. These results suggest the HiT T cells show improved function in a tumor microenvironment containing shed mesothelin compared with antibody-based receptors recognizing the same antigen.

### HiT T cells eradicate mesothelin-expressing solid tumors in vivo

**Fig 6:**
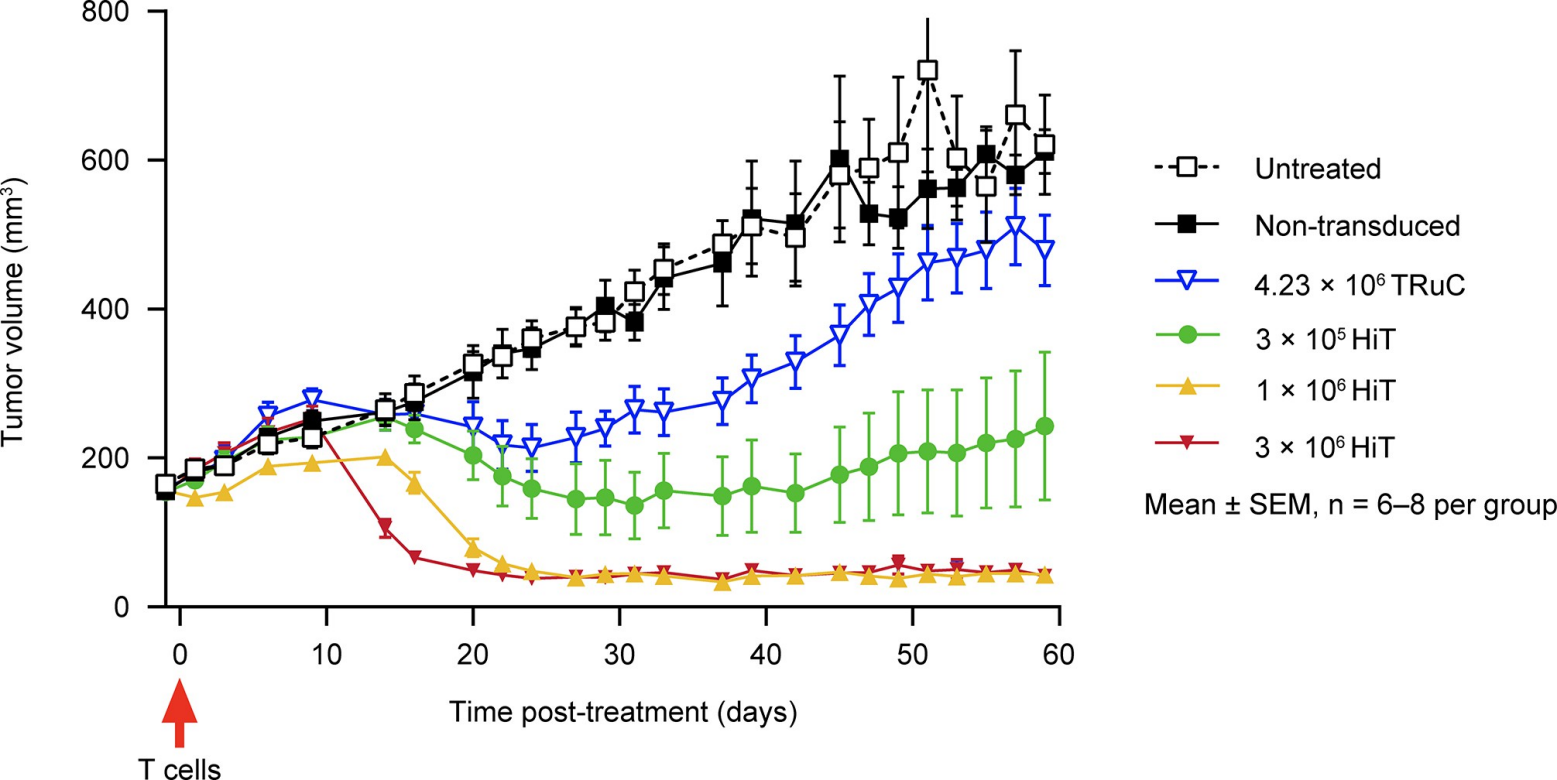
In vivo efficacy of parental mesothelin HiT T cells in a mouse xenograft solid tumor model. Human pancreatic Capan-2 tumor cells were inoculated subcutaneously with either non-transduced or transduced HiT or TRuC T cells (transduced dose as per legend) administered intravenously on day 0. Statistical analysis of these data is shown in S3 Table. HiT, human leukocyte antigen–independent T-cell receptor; SEM, standard error of the mean; TRuC, T-cell receptor fusion construct.

The in vivo antitumor efficacy of HiT T cells was evaluated in human pancreatic tumor cell line (Capan-2)-xenografted NSG mice. Because the mesothelin HiT does not bind to murine mesothelin (S6 Fig), no conclusion on safety can be reached from this study. HiT T cell–treated animals (n = 6–8) received 3 × 10^5^, 1 × 10^6^, or 3 × 10^6^ transduced T cells and a comparator group received a mesothelin TRuC at 4.23 × 10^6^ transduced T cells (Fig 6). Tumor-bearing mice treated with non-transduced T cells progressed at a similar rate to untreated mice. However, mice treated with HiT T cells demonstrated very robust, dose-dependent, and persistent antitumor efficacy, with full regression of tumors observed at doses ≥1 × 10^6^ T cells. Mice receiving TRuC T cells showed modest inhibition of tumor growth with 4.23 × 10^6^ T cells.

## Discussion

In the present study, we utilized our phage display libraries to isolate a TCR that binds in an HLA-independent manner directly to the tumor-associated antigen mesothelin. Testing of this HiT in cellular assays has demonstrated that it can bind to mesothelin displayed on the surface of target tumor cells and cause activation of the T cell, leading to cytotoxicity. We have further demonstrated that compared with a TRuC prepared in-house using our standard lentiviral vector and large-scale T-cell methods, this HiT is not inhibited by the presence of soluble mesothelin and appears much more potent than the TRuC in an in vivo model of pancreatic tumors, with full regression of the xenograft at doses of 1 million HiT T cells or greater. Although our HiT is more potent than the TRuC, it is important to note that on-target, off-tumor reactivity from a highly potent cell therapy may cause adverse events in a clinical setting. Currently, TRuC molecules are showing good clinical safety and efficacy [54] and any HiT will require the correct potency to deliver a safe therapeutic. To that end, we have shown here that we can fine-tune the potency of this HiT to recognize specific levels of target expression, and any clinical product will require tuning to the therapeutic window of the target.

This study is the first where it has been demonstrated that a TCR can be selected and engineered to induce T-cell killing against a specific tumor-associated antigen that is unrelated to HLA or similar proteins. Previous examples have recently been presented where the T-cell clone was first identified before elucidation of the binding partner and demonstration of HLA independence [10,12,13].

CARs utilizing antibody fragments fused to CD3ζ, with later generations also featuring co-stimulatory domains [55], have been used widely to target tumor-associated antigens. Although CARs have shown good clinical evidence of efficacy against hematologic cancers, challenges do still exist for the routine clinical use of CAR T-cell technologies. These challenges, which in part are due to the artificial basis of CAR T-cell signaling [55,56], include cytokine release syndrome and low efficacy against solid tumors. Additionally, the use of murine-derived antibody fragments has been shown to cause immune responses [57,58] and can require humanization of the antibody to combat this, whereas the use of co-stimulatory domains can lead to tonic signaling of the T cell, resulting in early exhaustion [59]. To overcome some of these issues, multiple formats have been developed to emulate the function of TCRs and engage the natural T-cell signaling pathways (Fig 1), including TRuCs in which antibody fragments are fused to proteins in the TCR:CD3 complex [60] and what we call variable fragment TCRs (FvTCRs), which replace the variable domains of the TCR with the light and heavy chain of an antibody [40]. The original authors termed these chimeric constructs HiTs, but we believe FvTCRs better reflect the antibody domain–based architecture. The use of fully human TCR phage display libraries and natural T-cell signaling, as found in HiTs, eliminates some of the issues facing CARs, TRuCs, and FvTCRs, and has been suggested to have great therapeutic utility [13].

The HiT binds to mesothelin with a much lower affinity than the antibody domains used in other cell therapies, such as TRuCs. This weaker affinity does not affect T-cell activation against membrane-bound mesothelin but results in less competition with soluble mesothelin compared with the high-affinity antibodies. Many targets of CAR T cells and similar therapeutics are expressed in soluble form or shed from the surface of tumor cells, and these soluble forms have been shown to inhibit CAR T-cell function [51,52]. Therefore, antigens that are highly shed and unsuitable for high-affinity antibody-based CAR T cells could potentially be targeted by HiT T cells or may remove the need for a co-therapy to inhibit antigen shedding, an approach currently being trialed for a B cell–specific antigen CAR-T (NCT03502577).

We have shown that our HiT-transduced T cells are activated in both the CD8 and CD4 subsets, which should be clinically beneficial in an autologous setting. Patient apheresis yields a mixture of both subsets, the proportions of which differ from patient to patient. TCR T-cell therapies targeting class I HLA can therefore contain CD4 T cells that do not fully activate in response to a signal through their introduced class I TCR, although this can be overcome by the addition of *CD8A* to the transgene added to the T cells (NCT04044859). A HiT T-cell product would not require this additional transgene, freeing capacity in the lentiviral vector for the addition of other next-generation elements.

Taken together, we have shown, for the first time, the ability to isolate a HiT against a specific target and then modulate the binding, activation, and cytotoxic characteristics of HiTs, an important step toward the development of a safe therapeutic product with limited on-target, off-tumor toxicity. If this HiT were to move toward first-in-human trials, significant preclinical testing would be required to ensure that no cross-reactivity to other molecules, especially pHLA complexes, exist potentially due to the lack of thymic selection this HiT has experienced. This preclinical testing falls outside of the scope of this proof-of-concept manuscript. Given the efficacy of HiT T cells against solid tumors in vivo, we believe that HiTs using natural T-cell signaling have the potential to address many of the limitations facing the field of CAR T-cell therapy, as well as allowing for wider patient access and a large proportion of efficacious T cells compared with traditional TCR T-cell therapies.

## Supporting information

S1 Methods (DOCX)

S1 Table Levels of mesothelin mRNA for a panel of cell lines, as quantified by quantitative polymerase chain reaction. (DOCX)

S2 Table Transduction efficiencies of T cells used throughout the manuscript where not featured directly in figures. Efficiencies measured by flow cytometry using specific Vbeta antibodies for TCRs or CD3 expression in the case of 5B or mesothelin tetramer for the TRuC. HiT, human leukocyte antigen–independent T-cell receptor; Mut, mutant; TCR, T-cell receptor; TRuC, T-cell receptor fusion construct; WT, wild type.
(DOCX)

S3 Table Statistical analysis of in vivo data shown in Fig 6. Data were analyzed using either a repeated measures two-way analysis of variance or a mixed-effects model with Geisser-Greenhouse correction (GraphPad Prism) when there were values missing from the data due to early termination of animals from the study (indicated with asterisks). HiT, human leukocyte antigen–independent T-cell receptor; TCR, T-cell receptor; TRuC, T-cell receptor fusion construct.
(DOCX)

S1 Fig Example of gating strategy for CD69 activation assay for non-transduced Jurkat cells. CD69-positive events were quantified upon gating on lymphocytes (SSC-A vs FSC-A), singlets (FSC-A vs FSC-H), live cells (7-AAD vs FSC-A), and CD8- and CD3-positive cells.
(TIF)

S2 Fig Representative example of surface plasmon resonance data for the wild-type mesothelin human leukocyte antigen–independent T-cell receptor at a range of concentrations binding to human mesothelin (296–580). (TIF)

S3 Fig Surface plasmon resonance data and equilibrium binding with a fit to a 1:1 Langmuir equation for T-cell receptor binding to truncated mesothelin constructs. Concentrations of each curve of the sensogram corresponds to the points in the adjacent equilibrium binding curves. Sensograms for truncated mesothelin constructs are not shown if no binding was detected. (A) M5 (296–524), (B) M6 (296–566), (C) M7 (296–580), (D) MN1 (391–580), (E) MN2 (439–580), and (F) MN3 (469–580). K_D_, equilibrium dissociation constant; ND, not determined; T_1/2_, dissociation half-life.
(TIF)

S4 Fig Staining of cell lines with W6/32-APC (BioLegend) to detect HLA class I. K562 (orange) shows detectable expression of HLA class I, whereas both DLD1 (red) and SNG-M (green) show no detectable expression of HLA class I. HLA, human leukocyte antigen.
(TIF)

S5 Fig Activity of parental mesothelin HiT and a panel of eight mutants in a Jurkat activation assay. Transduction was measured by CD3 expression (purple) of T cells when cells were cultured alone. Activation of HiT T cells, measured by percentage of CD69-positive transduced Jurkat T cells, when co-cultured with antigen-positive HCT116 (green) and antigen-negative C32TG (red) cells or when cultured alone (blue). Assay conditions were prepared in duplicate with individual points represented by a single circle. HiT, human leukocyte antigen–independent T-cell receptor; WT, wild type.
(TIF)

S6 Fig Example of surface plasmon resonance data of HiT and antibody binding to human and murine mesothelin. The HiT can be seen to bind to human mesothelin (yellow) but not murine mesothelin (green), whereas the control antibody can be seen to bind to both human mesothelin (red) and murine mesothelin (blue). HiT, human leukocyte antigen–independent T-cell receptor.
(TIF)

S1 Data Alpha and beta variable domains of the mesothelin HiT. (DOCX)
