# Supplementary material for: Selection, engineering, and in vivo testing of a human leukocyte antigen–independent T-cell receptor recognizing human mesothelin
Source: PLoS One. 2024 Apr 4;19(4):e0301175. doi: 10.1371/journal.pone.0301175 (PMC10994368; doi:10.1371/journal.pone.0301175)
Supplement: S1 Methods — (DOCX) [file pone.0301175.s001.docx]

**S1 Methods**

**Production of recombinant mesothelin**

For human leukocyte antigen–independent T-cell receptor (HiT) and T-cell receptor fusion construct (TRuC) blocking experiments, soluble human recombinant mesothelin (E296-G580, Q13421-3, labeled as M7 for epitope analysis with C-terminal Avi and decaHis tags) was produced from adherent HEK293T cells by transfection of a mesothelin-encoding plasmid along with a plasmid encoding BirA for biotinylation, using TurboFect (Thermo Fisher Scientific, R0532), according to the manufacturer’s instructions. Cell culture supernatant was collected and purified using, first, immobilized metal chelate affinity chromatography using a HisTrap HP 5 mL (GE Healthcare) column, followed by size exclusion chromatography with a Superdex 200 Increase 10/300 GL (GE Healthcare) column. For epitope analysis, truncated mesothelin constructs M1 (E296-L359), M2 (E296-V401), M3 (E296-Y437), M4 (E296-F484), M5 (E296-M524), and M6 (E296-Q566) were produced in adherent HEK293T as above. Truncated mesothelin constructs MN1 (S391-G580), MN2 (G349-G580), MN3 (D469-G580), and MN4 (N488-G580) were also produced from adherent HEK293T, but without biotinylation. Cell culture supernatants were collected, and buffer exchanged by repeated concentration and dilution using Vivaspin Turbo 4 (Sartorius).

**Lentivirus production**

For small-scale lentivirus production linked to Jurkat assays, HEK293T cells adhered to six-well plates (VWR) were transfected with lentiviral plasmids encoding the T-cell antigen receptor (TCR) sequence of interest and packaging plasmid mix (Puresyn/Aldevron). Cells were incubated for 48 h at 37°C under 5% CO_2_, after which virus-containing supernatants were removed for transduction.

For large-scale lentivirus production linked to primary T-cell assays, HEK293T cells in suspension culture were transfected with lentiviral plasmids encoding the TCR sequence of interest and packaging plasmid mix (Puresyn/Aldevron). Cultures were supplemented with 500 mM sodium butyrate after 24 h. Lentiviral supernatants were harvested after 48 h in culture and clarified by centrifugation in TubeSpin bioreactor tubes (VWR) and syringe filtration.

**Stable transfection of mesothelin plasmid**

K562 and A375 cells were transfected with the commercially available pcDNA3.4 plasmid (Thermo Fisher Scientific) that contained a cytomegalovirus promoter driving mesothelin transcript variant 3, followed by a P2A site and green fluorescent protein, in the multiple cloning site. The transfection was carried out with TurboFect (R0532, Thermo Fisher Scientific), per the manufacturer’s instructions. After 3 weeks of culture in standard medium supplemented with G418 (Roche Diagnostics; 0.6 mg/mL), transfection was validated by flow cytometry for the presence of green fluorescent protein.

**Quantitative polymerase chain reaction assay**

Total RNA was extracted using the Maxwell RSC simplyRNA Cells Kit (Promega) and cDNA was prepared using the qScript cDNA SuperMix (Quantabio), according to the manufacturer’s protocol. Subsequent quantitative polymerase chain reaction (qPCR) was carried out on the QuantStudio 7 Real-Time PCR System (Thermo Fisher Scientific) using the QuantiTect Probe PCR Kit (Qiagen) and TaqPath qPCR Master Mix (Thermo Fisher Scientific).

Gene expression was quantified by comparing the cycle threshold values of the test samples to a standard curve with known number of copies (plasmid). Data are represented following normalization to the average of a high (*RPL32*) and low (*HPRT1*) expression housekeeping gene. The combination was chosen from 38 housekeeping genes because it generated the most stable results in a panel of 95 different samples, including immune cells.

**Supernatant enzyme-linked immunosorbent assays**

Human interferon γ (IFN-γ) DuoSet and Human Granzyme B DuoSet enzyme-linked immunosorbent assay (ELISA) kits (DY285B and DY2906-05, respectively, R&D Systems) were used to assess IFN-γ and granzyme B release, respectively. Manufacturer protocols were followed, with these minor modifications: 3% bovine serum albumin (BSA)/phosphate-buffered saline (PBS) solution was used for the granzyme B ELISA blocking step. Standard curve ranges used for cell lines assays were 19.5–5,000 pg/mL (IFN-γ) and 19.5–10,000 pg/mL (granzyme B), whereas 9.4–2,400 pg/mL (IFN-γ) and 39.1–10,000 pg/mL (granzyme B) were used for patient-derived xenograft (PDX) samples. Supernatants were diluted three-fold in reagent diluent (PBS/1% BSA); supernatants were not diluted for PDX assays.

**Capan-2 xenograft model**

Mice (NSG male, 6–7 weeks of age, body weight ≥15 g) were randomized based on mean tumor volume, 10 days after subcutaneous inoculation with 1 × 10^7^ tumor cells in Matrigel (100 µL), using study management software (Studylog Systems, Pacifica, CA, USA). Mean tumor volume for each group at randomization was approximately 157.71 mm^3^. Sample size was determined based on published guidelines [1] and the authors’ prior experience with tumor cell lines in vivo. Laboratory scientists were unaware of the target antigen of the administered T cells.

- Group 1: untreated; n = 7
- Group 2: non-transduced T cells (3 × 10^6^); n = 8
- Group 3: HiT (3 × 10^5^); n = 8
- Group 4: HiT (1 × 10^6^); n = 8
- Group 5: HiT (3 × 10^6^); n = 8
- Group 6: TruC (3 × 10^6^); n = 8

Mice were housed in individually ventilated cages and acclimatization occurred for ≥7 days. Water and food (Teklad 2919) were consumed ad libitum.

Mice were terminated early if their body weight decreased by ≥20% or >15% for three consecutive measurements (compared with day 1 treatment), if mean tumor diameter was ≥15 mm, or if any adverse clinical signs or loss of clinical condition (e.g., tumor ulceration) were observed. T-cell administration was well tolerated, with mice gaining weight throughout the study; however, a total of 12 mice were terminated early due to tumor ulceration or tumors surpassing allowable size.

Mean tumor volume was analyzed statistically using either a repeated measures two-way analysis of variance or a mixed-effects model with Geisser-Greenhouse correction (GraphPad Prism, GraphPad Software Inc., San Diego, CA, USA) when there were values missing from the data due to early termination of animals from the study. Body weights were measured daily for 16 days from day –1 (randomization), and on the same day as the tumor volume measurements thereafter. Tumor volumes (mm^3^) were measured at least three times per week. The same operator performed all tumor measurements throughout the duration of the study to ensure consistency.

**Reference**

**1.** Workman P, Aboagye EO, Balkwill F, Balmain A, Bruder G, Chaplin DJ, et al. Guidelines for the welfare and use of animals in cancer research. Br J Cancer. 2010; 102: 1555–1577. https://doi.org/10.1038/sj.bjc.6605642 PMID: 20502460.
