# Supplementary material for: Selection, engineering, and in vivo testing of a human leukocyte antigen–independent T-cell receptor recognizing human mesothelin
Source: PLoS One. 2024 Apr 4;19(4):e0301175. doi: 10.1371/journal.pone.0301175 (PMC10994368; doi:10.1371/journal.pone.0301175)
Supplement: S1 Table — (DOCX) [file pone.0301175.s002.docx]

**S1 Table. Levels of mesothelin** **mRNA for a panel of cell lines, as quantified by quantitative polymerase chain reaction.**

| **Target cells** | **Indication/type (supplier)** | **Culture maintenance medium** | **Level of mesothelin mRNA^a^** | **HLA class I allotypes^b^** | **HLA class II allotypes^c^** | **Other comments** |
| --- | --- | --- | --- | --- | --- | --- |
| Capan-2 | Pancreatic adenocarcinoma cell line (ATCC) | M10 | 313,438.7 | A*29:01 A*29:01 B*44:03 B*44:03 C*16:01 C*16:01 | DQA1*02:01 DQA1*02:01  DQB1*02:02 DQB1*06:11 DRB1*07:01 DRB1*14:05 |  |
| SNG-M | Endometrial adenocarcinoma cell line (JRCB) | H20 | 55,629.4 | A*11:01 A*26:03 B*54:01 B*51:01 C*01:02 C*03:04 | DQB1*05:02 DQB1*05:02 | No surface HLA class I expression |
| HCT116 | Colorectal cancer cell line (ATCC) | M10 | 21,673.0 | A*01:01 A*02:01 B*18:01 B*45:01 C*05:01 C*07:01 | DQB1*02:02 DQB1*02:02 DRB1*03:05 DRB1*03:05 |  |
| HeLa | Cervical adenocarcinoma cell line (ATCC) | E10 | 11,582.7 | A*68:02 A*68:02 B*15:03 B*15:03 C*12:03 C*12:03 | DQA1*01:02 DQA1*01:02  DQB1*05:01 DQB1*05:01 DRB1*01:02 DRB1*01:02 |  |
| Capan-1 | Pancreatic Adenocarcinoma cell line (ATCC) | I20 | 11,110.3 | A*01:01 A*30:01 B*13:02 B*57:01 C*06:02 C*06:02 | DQA1*02:01 DQA1*02:01  DQB1*02:02 DQB1*06:11 DRB1*07:01 DRB1*14:05 |  |
| HepG2 | Hepatocellular carcinoma cell line (ATCC) | E10 | 4,799.4 | A*02:01 A*24:02 B*35:14 B*51:08 C*04:01 C*16:02 | DQB1*06:04 DQB1*06:04 |  |
| DLD-1 | Colorectal adenocarcinoma cell line (ECACC) | R10 | 2,401.7 | A*02:01 A*24:02 B*35:01 B*08:01 C*04:01 C*07:01 |  | No surface HLA class I expression |
| DLD-1.A2β2m | Colorectal adenocarcinoma cell line (ECACC) | R10 | 1,565.2 | A*02:01 A*24:02 B*35:01 B*08:01 C*04:01 C*07:01 |  |  |
| A375 | Melanoma cell line (ATCC) | R10 | 507.3 | A*01:01 A*02:01 B*44:03 B*57:01 C*06:02 C*16:01 | DQA1*03:02 DQA1*02:01  DQB1*03:02 DQB1*03:02 DRB1*04:05 DRB1*07:01 |  |
| Cama-1 | Breast adenocarcinoma cell line (ATCC) | E10 | 342.9 | A*02:01 A*32:01 B*15:01 B*40:02 C*02:02 C*03:03 | DQA1*05:02 DQA1*03:02 DQB1*03:04 DQB1*03:04 DRB1*11:30 DRB1*13:07 |  |
| SK-BR-3 | Breast adenocarcinoma cell line (ATCC) | M10 | 80.6 | A*02:01 A*11:01 B*35:21 B*14:02 C*03:04 C*03:04 | DQB1*06:04 DQB1*06:04 DRB1*01:05 DRB1*01:05 |  |
| K562 | Chronic myelogenous leukemia cell line (ATCC) | I10 | 17.6 | A*24:02 A*26:02 B*81:01 B*35:186 C*05:01 C*03:04 |  | Possibly only HLA-C on surface |
| Lung 1 | NSCLC PDX (Crown Bioscience) | ACL-4 | 752,788 | A*01:01 A*02:01 B*35:02 B*57:01 C*04:01 C*06:02 |  |  |
| Lung 2 | NSCLC PDX (Crown Bioscience) | ACL-4 | 366,620 | A*02:01 A*02:01 B*07:02 B*27:05 C*01:02 C*07:02 |  |  |

^a^Normalized per 10^6^ reference gene transcripts.

^b^From TRON Cell Line Portal, except for PDX models - from supplier RNAseq data [2].

^c^From TRON Cell Line Portal, where available [2].

ACL-4, Advanced DMEM/F12 + 0.1 μM Hydrocortisone + 60 μg/ml Bovine Pituitary extract + 4 mM L-glutamine + 2% v/v Pen/Strep + 20 ng/ml EGF + 10 μM Y-27632; ATCC, American Type Culture Collection; E10, EMEM + 10% v/v FBS + 1% v/v Pen/Strep; ECAC, European Collection of Authenticated Cell Cultures;H20, Ham’s F12 medium + 20% v/v FBS + 1% v/v Pen/Strep; HLA, human leukocyte antigen; I10, IMDM + 10% v/v FBS + 1% v/v Pen/Strep; I20, IMDM + 20% v/v FBS + 1% v/v Pen/Strep; JRCB, Japanese Collection of Research Biosources Cell Bank; M10, McCoy’s 5A medium + 10% v/v FBS + 1% v/v Pen/Strep; mRNA, messenger RNA; NSCLC, non–small cell lung cancer; PDX, patient-derived xenograft; R10, RPMI 1640 + 10% v/v FBS + 1% v/v Pen/Strep.

Reference: 2. Scholtalbers J, Boegel S, Bukur T, Byl M, Goerges S, Sorn P, et al. TCLP: an online cancer cell line catalogue integrating HLA type, predicted neo-epitopes, virus and gene expression. Genome Med. 2015; 7: 118. https://doi.org/10.1186/s13073-015-0240-5 PMID: 26589293.
