# Supplementary material for: Selection, engineering, and in vivo testing of a human leukocyte antigen–independent T-cell receptor recognizing human mesothelin
Source: PLoS One. 2024 Apr 4;19(4):e0301175. doi: 10.1371/journal.pone.0301175 (PMC10994368; doi:10.1371/journal.pone.0301175)
Supplement: S2 Table — Efficiencies measured by flow cytometry using specific Vbeta antibodies for TCRs or CD3 expression in the case of 5B or mesothelin tetramer for the TRuC. HiT, human leukocyte antigen–independent T-cell receptor; Mut, mutant; TCR, T-cell receptor; TRuC, T-cell receptor fusion construct; WT, wild type. (DOCX) [file pone.0301175.s003.docx]

**S2 Table. Transduction efficiencies of T cells used throughout the manuscript where not featured directly in figures.** Efficiencies measured by flow cytometry using specific Vbeta antibodies for TCRs or CD3 expression in the case of 5B or mesothelin tetramer for the TRuC.

| **Figure** | **T cell** | **Transduction efficiency, %** |
| --- | --- | --- |
| 3A–F | HiT | 51.4 |
| 3G–J | HiT | 60.2 |
| 4 | Mut1 | 52.7 |
| 4 | Mut2 | 59.8 |
| 4 | Mut3 | 57.8 |
| 4 | Mut4 | 55 |
| 4 | Mut5 | 36.5 |
| 4 | WT | 60.2 |
| 4 | Mut6 | No data available |
| 4 | Mut7 | 37.9 |
| 4 | Mut8 | 60.9 |
| 5A | HiT | 48.9 |
| 5A | TRuC | 50 |
| 5A | TCR | 30.1 |
| 5B | HiT | 96.7 |
| 5B | TRuC | 92.3 |
| 6 | HiT | 61.6 |
| 6 | TRuC | 70.5 |

HiT, human leukocyte antigen–independent T-cell receptor; Mut, mutant; TCR, T-cell receptor; TRuC, T-cell receptor fusion construct; WT, wild type.
