# Supplementary material for: Selection, engineering, and in vivo testing of a human leukocyte antigen–independent T-cell receptor recognizing human mesothelin
Source: PLoS One. 2024 Apr 4;19(4):e0301175. doi: 10.1371/journal.pone.0301175 (PMC10994368; doi:10.1371/journal.pone.0301175)
Supplement: S3 Table — Data were analyzed using either a repeated measures two-way analysis of variance or a mixed-effects model with Geisser-Greenhouse correction (GraphPad Prism) when there were values missing from the data due to early termination of animals from the study (indicated with asterisks). HiT, human leukocyte antigen–independent T-cell receptor; TCR, T-cell receptor; TRuC, T-cell receptor fusion construct. (DOCX) [file pone.0301175.s004.docx]

**S3 Table. Statistical analysis of in vivo data shown in Fig 6.** Data were analyzed using either a repeated measures two-way analysis of variance or a mixed-effects model with Geisser-Greenhouse correction (GraphPad Prism) when there were values missing from the data due to early termination of animals from the study (indicated with asterisks).

|  | **Non-transduced** | **3 × 10^5^ HiT** | **1 × 10^6^ HiT** | **3 × 10^6^ HiT** | **3 × 10^6^ TRuC** |
| --- | --- | --- | --- | --- | --- |
| Untreated | p = 0.9915* | p < 0.0001* | p < 0.0001* | p < 0.0001* | p < 0.0001* |
| Non-transduced |  | p < 0.0001* | p < 0.0001* | p < 0.0001* | p < 0.0001* |
| 3 × 10^5^ HiT |  |  | p < 0.0001 | p < 0.0001 | p < 0.0001 |
| 1 × 10^6^ HiT |  |  |  | p < 0.0001 | p < 0.0001 |
| 3 × 10^6^ HiT |  |  |  |  | p < 0.0001 |

HiT, human leukocyte antigen–independent T-cell receptor; TRuC, T-cell receptor fusion construct.
