# Supplementary figures and images for: Selection, engineering, and in vivo testing of a human leukocyte antigen–independent T-cell receptor recognizing human mesothelin
Source: PLoS One. 2024 Apr 4;19(4):e0301175. doi: 10.1371/journal.pone.0301175 (PMC10994368; doi:10.1371/journal.pone.0301175)

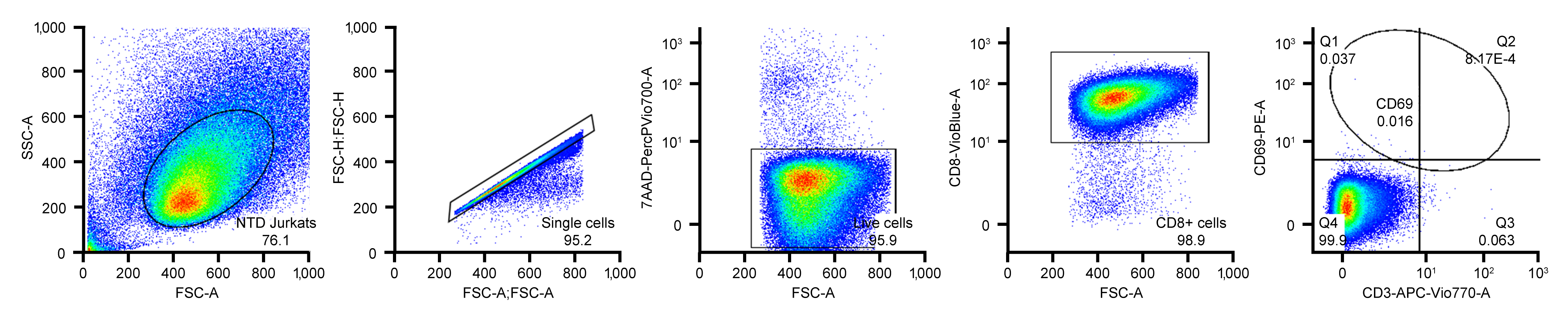

Supplement: S1 Fig — CD69-positive events were quantified upon gating on lymphocytes (SSC-A vs FSC-A), singlets (FSC-A vs FSC-H), live cells (7-AAD vs FSC-A), and CD8- and CD3-positive cells. (TIF) [file pone.0301175.s005.tif]

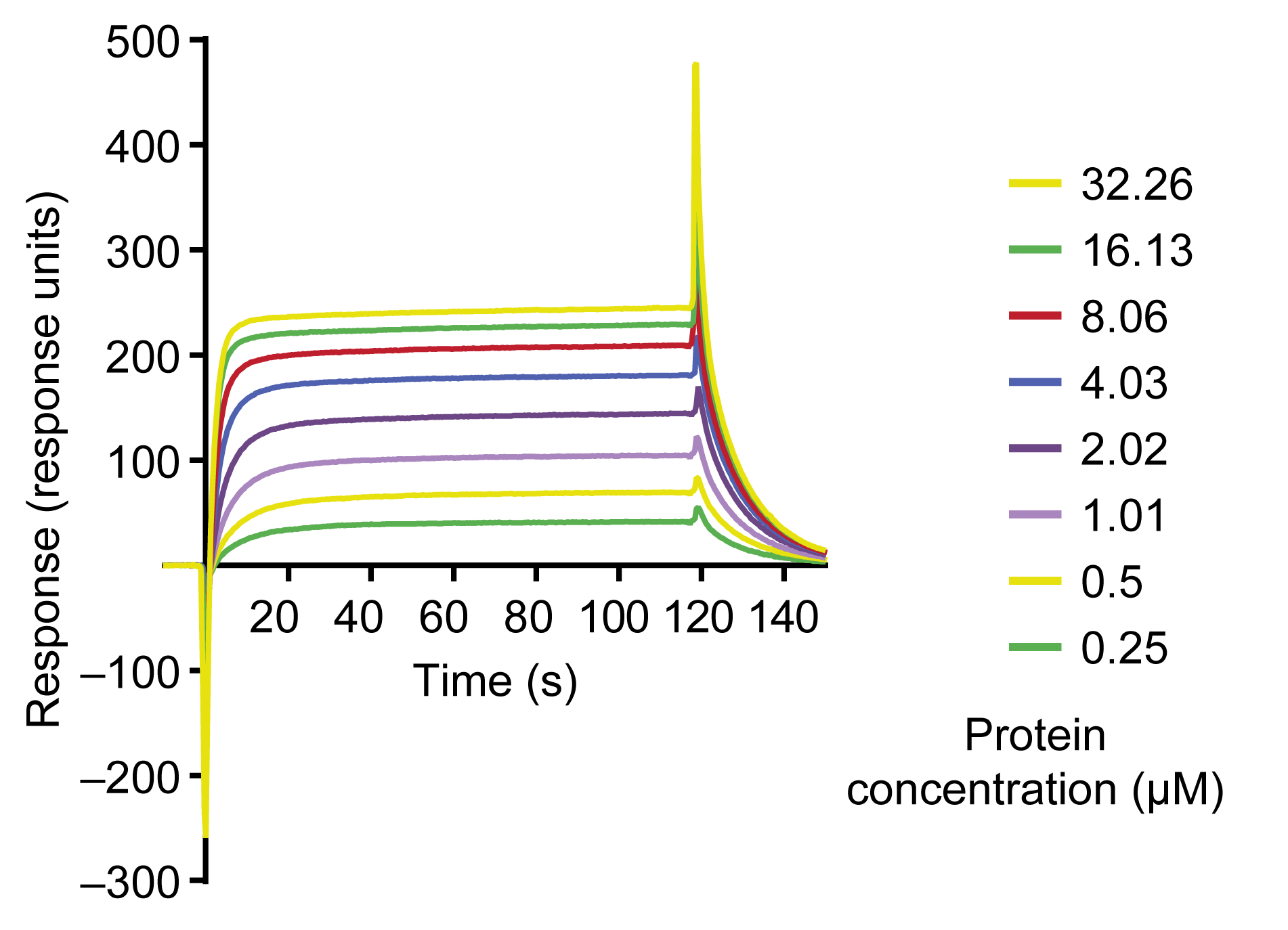

Supplement: S2 Fig — (TIF) [file pone.0301175.s006.tif]

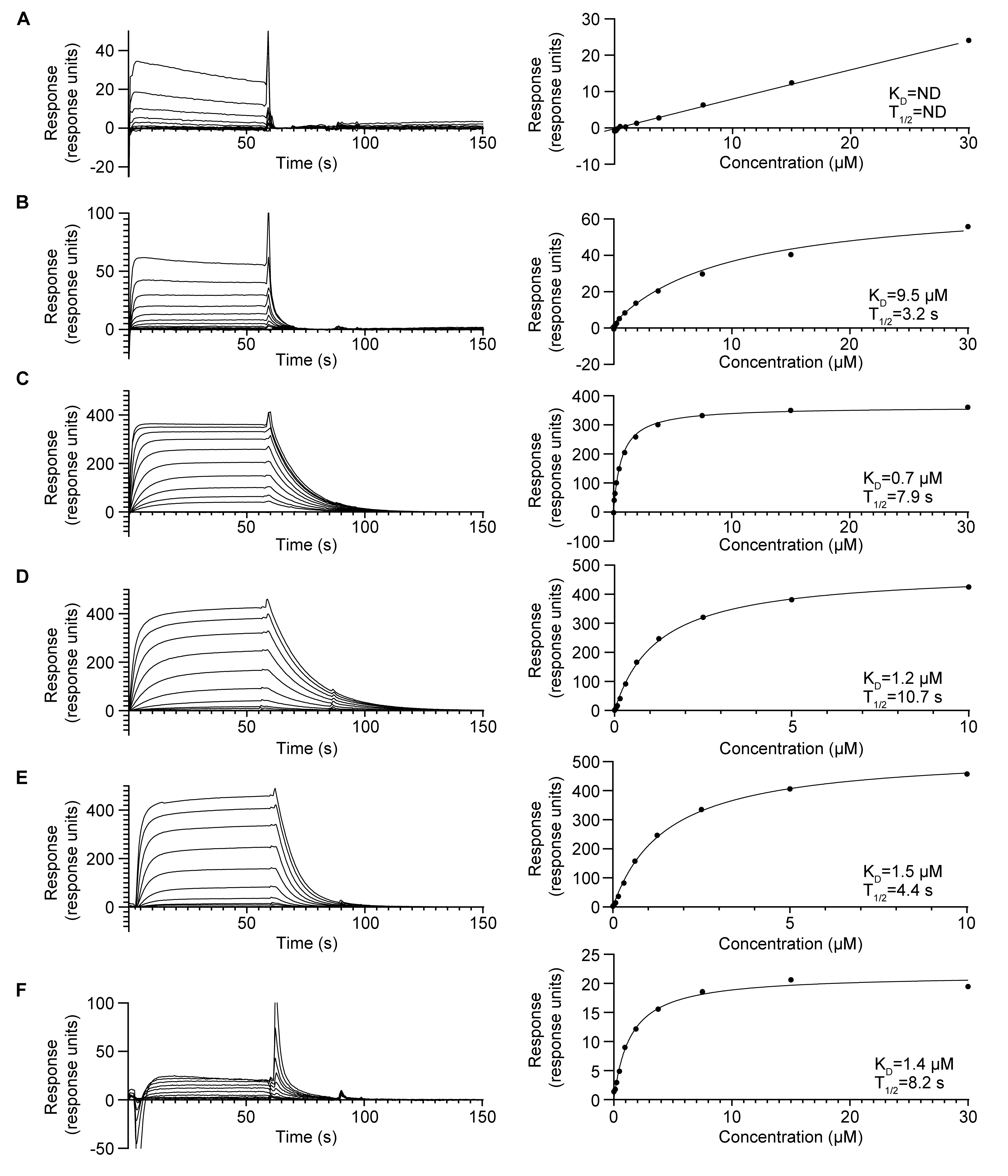

Supplement: S3 Fig — Concentrations of each curve of the sensogram corresponds to the points in the adjacent equilibrium binding curves. Sensograms for truncated mesothelin constructs are not shown if no binding was detected. (A) M5 (296–524), (B) M6 (296–566), (C) M7 (296–580), (D) MN1 (391–580), (E) MN2 (439–580), and (F) MN3 (469–580). KD, equilibrium dissociation constant; ND, not determined; T1/2, dissociation half-life. (TIF) [file pone.0301175.s007.tif]

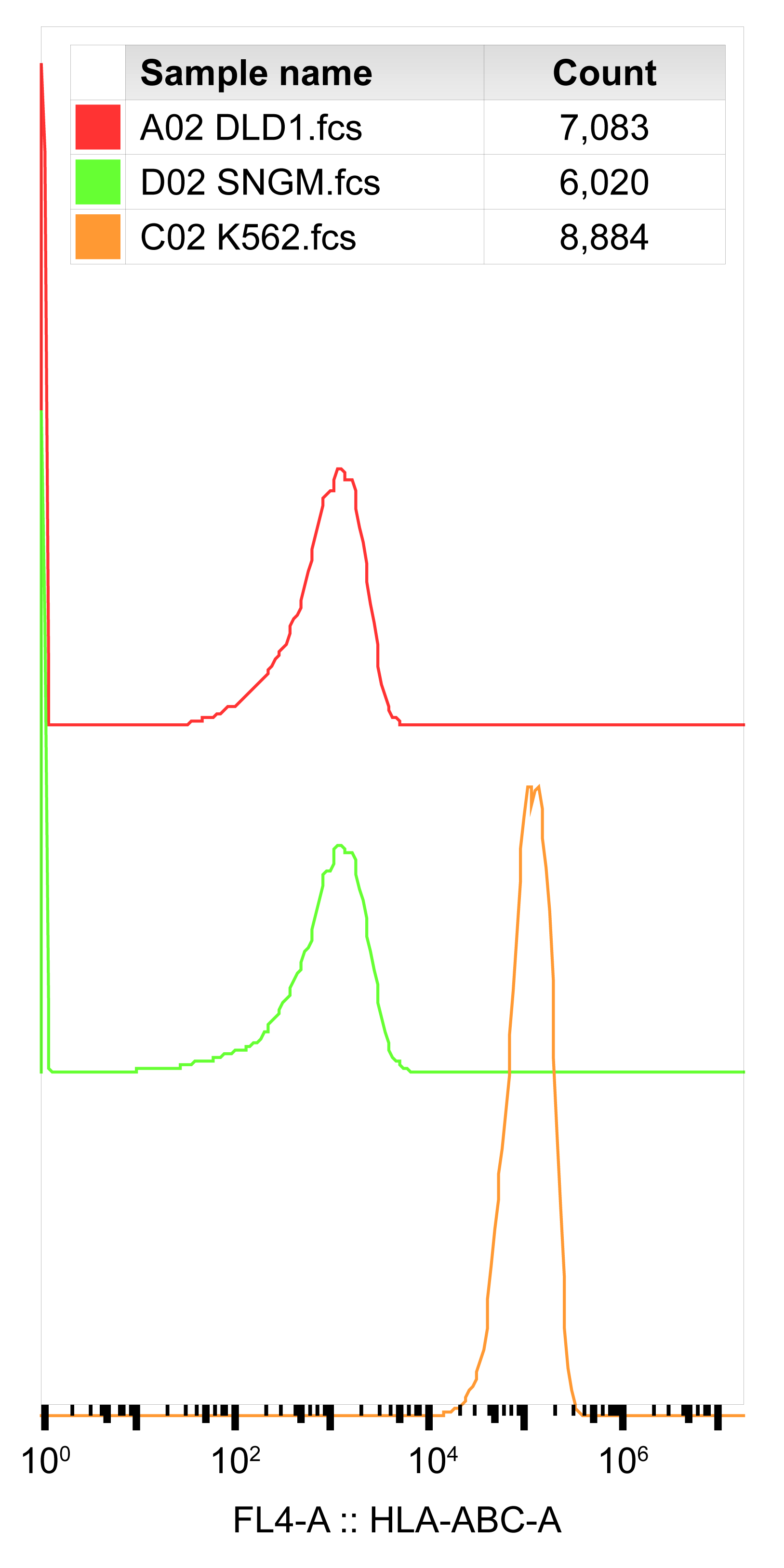

Supplement: S4 Fig — K562 (orange) shows detectable expression of HLA class I, whereas both DLD1 (red) and SNG-M (green) show no detectable expression of HLA class I. HLA, human leukocyte antigen. (TIF) [file pone.0301175.s008.tif]

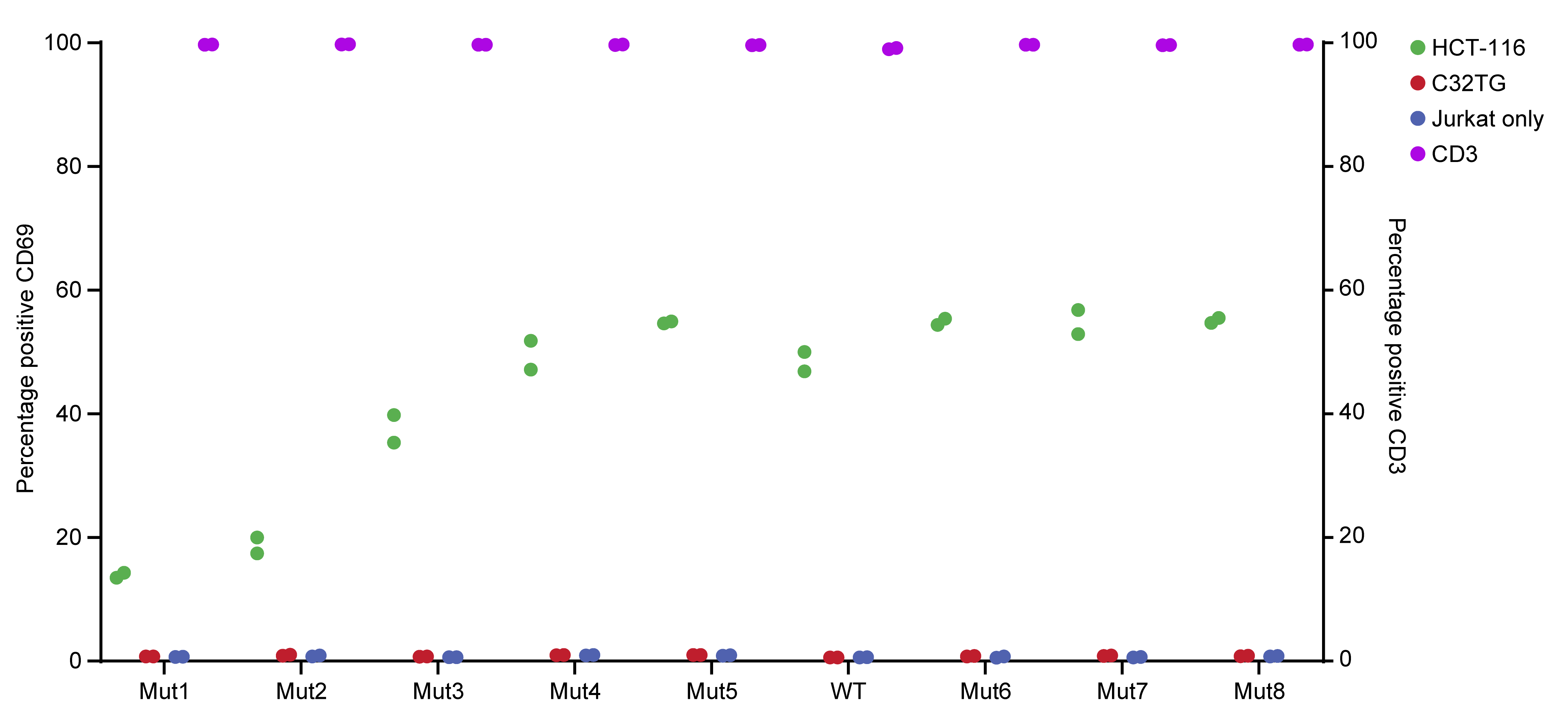

Supplement: S5 Fig — Transduction was measured by CD3 expression (purple) of T cells when cells were cultured alone. Activation of HiT T cells, measured by percentage of CD69-positive transduced Jurkat T cells, when co-cultured with antigen-positive HCT116 (green) and antigen-negative C32TG (red) cells or when cultured alone (blue). Assay conditions were prepared in duplicate with individual points represented by a single circle. HiT, human leukocyte antigen–independent T-cell receptor; WT, wild type. (TIF) [file pone.0301175.s009.tif]

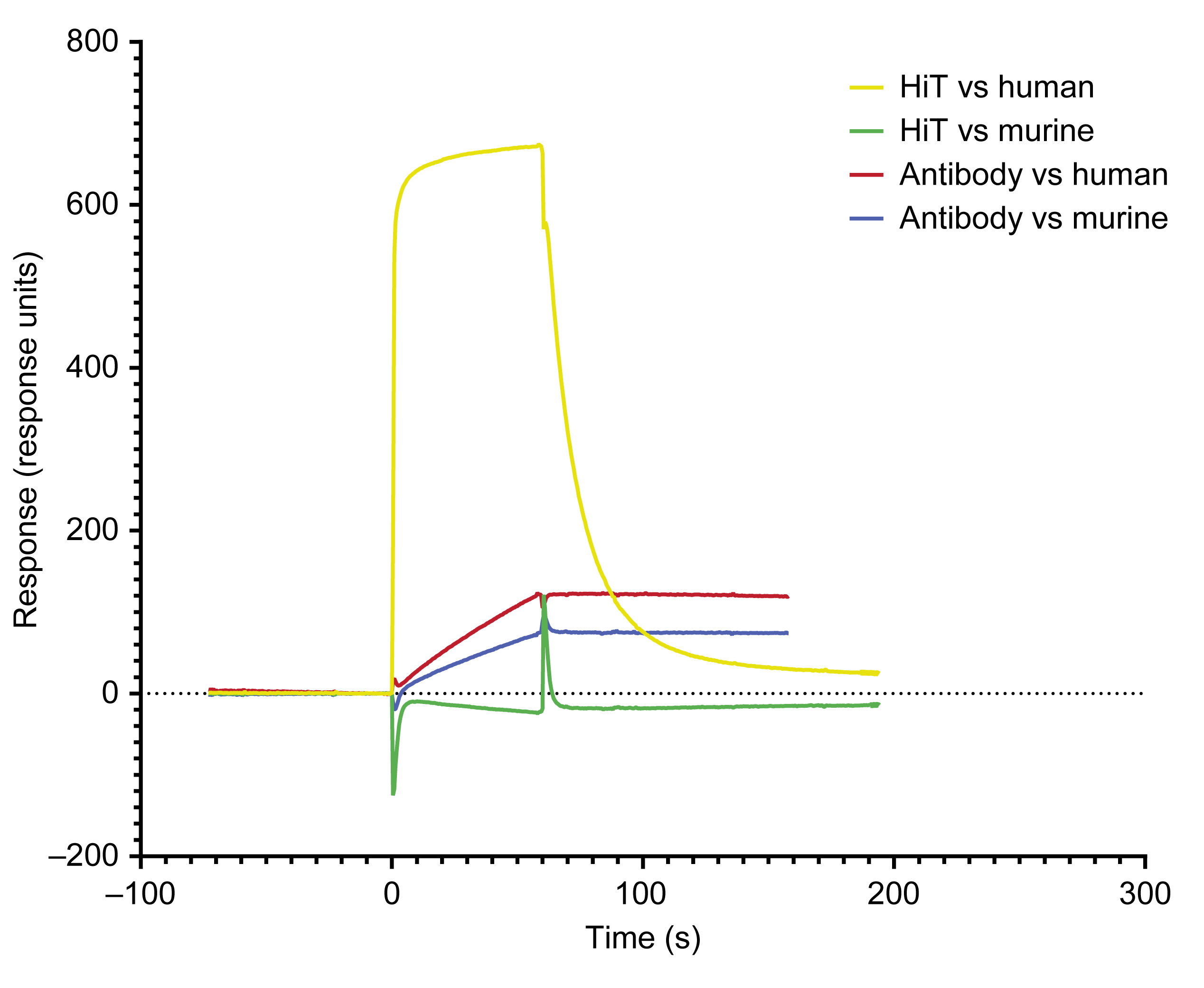

Supplement: S6 Fig — The HiT can be seen to bind to human mesothelin (yellow) but not murine mesothelin (green), whereas the control antibody can be seen to bind to both human mesothelin (red) and murine mesothelin (blue). HiT, human leukocyte antigen–independent T-cell receptor. (TIF) [file pone.0301175.s010.tif]
