# Supplementary material for: Selection, engineering, and in vivo testing of a human leukocyte antigen–independent T-cell receptor recognizing human mesothelin
Source: PLoS One. 2024 Apr 4;19(4):e0301175. doi: 10.1371/journal.pone.0301175 (PMC10994368; doi:10.1371/journal.pone.0301175)
Supplement: S1 Data — (DOCX) [file pone.0301175.s011.docx]

**S1 Data.** Alpha and beta variable domains of the mesothelin HiT.

QKEVEQNSGPLSVPEGAIASLNCTYSDRGSQSFFWYRQYSGKSPELIMSIYSNGDKEDGRFTAQLNKASQYVSLLIRDSQPSDSATYLCAVNRNRDDKIIFGKGTRLHILPN

NAGVTQTPKFGVLKTGQSMTLLCAQDMNHEYMYWYRQDPGMGLRLIHYSVGEGTTAKGEVPDGYNVSRLKKQNFLLGLESAAPSQTSVYFCASTAFWGGAEAFFGQGTRLTVVE
